# Risk factors for graft-versus-host-disease after donor lymphocyte infusion following T-cell depleted allogeneic stem cell transplantation

**DOI:** 10.3389/fimmu.2024.1335341

**Published:** 2024-03-13

**Authors:** Eva A. S. Koster, Peter A. von dem Borne, Peter van Balen, Erik W. A. Marijt, Jennifer M. L. Tjon, Tjeerd J. F. Snijders, Daniëlle van Lammeren, Hendrik Veelken, J. H. Frederik Falkenburg, Constantijn J. M. Halkes, Liesbeth C. de Wreede

**Affiliations:** ^1^ Department of Hematology, Leiden University Medical Center, Leiden, Netherlands; ^2^ Department of Hematology, Medical Spectrum Twente, Enschede, Netherlands; ^3^ Department of Hematology, HagaZiekenhuis, The Hague, Netherlands; ^4^ Department of Biomedical Data Sciences, Leiden University Medical Center, Leiden, Netherlands

**Keywords:** allogeneic stem cell transplantation, donor lymphocyte infusion, graft-versus-host-disease, acute myeloid leukemia, acute lymphoblastic leukemia, myelodysplastic syndrome, multi-state modelling

## Abstract

**Introduction:**

Unmodified donor lymphocyte infusions (DLI) after allogeneic stem cell transplantation (alloSCT) can boost the beneficial Graft-versus-Leukemia (GvL) effect but may also induce severe Graft-versus-Host-Disease (GvHD). To improve the balance between GvL and GvHD, it is crucial to identify factors that influence the alloreactivity of DLI.

**Methods:**

We investigated the effects of the presence of patient-derived antigen-presenting cells at time of DLI as estimated by the bone marrow (BM) chimerism status, lymphopenia as measured by the absolute lymphocyte count (ALC) at time of DLI, and the presence of a viral infection (*de novo* or reactivation) close to DLI on the risk of GvHD after DLI. The cohort consisted of patients with acute leukemia or myelodysplastic syndrome who prophylactically or pre-emptively received DLI as standard care after alemtuzumab-based alloSCT. In patients at high risk for relapse, DLI was administered at 3 months after alloSCT (n=88) with a dose of 0.3x10^6^ or 0.15x10^6^ T cells/kg in case of a related or unrelated donor, respectively. All other patients (n=76) received 3x10^6^ or 1.5x10^6^ T cells/kg, respectively, at 6 months after alloSCT.

**Results:**

For both DLIs, patients with reduced-intensity conditioning and an unrelated donor had the highest risk of GvHD. For DLI given at three months, viral infection within 1 week before and 2 weeks after DLI was an additional significant risk factor (hazard ratio (HR) 3.66 compared to no viral infection) for GvHD. At six months after alloSCT, viral infections were rare and not associated with GvHD. In contrast, mixed BM chimerism (HR 3.63 for ≥5% mixed chimerism compared to full donor) was an important risk factor for GvHD after DLI given at six months after alloSCT. ALC of <1000x10^6^/l showed a trend for association with GvHD after this DLI (HR 2.05 compared to ≥1000x106/l, 95% confidence interval 0.94-4.45). Furthermore, the data suggested that the presence of a viral infection close to the DLI at three months or ≥5% mixed chimerism at time of the DLI at six months correlated with the severity of GvHD, thereby increasing their negative impact on the current GvHD-relapse-free survival.

**Conclusion:**

These data demonstrate that the risk factors for GvHD after DLI depend on the setting of the DLI.

## 1 Introduction

The Graft-versus-Leukemia (GvL) effect of allogeneic hematopoietic stem cell transplantation (alloSCT) results from elimination of persisting malignant hematopoietic cells by donor-derived alloreactive T cells (1). The GvL effect can provide enduring relapse-free survival but can be accompanied by Graft-versus-Host-Disease (GvHD) when non-hematopoietic cells are targeted (2). T-cell depletion (TCD) reduces the risk of severe GvHD, but increases the relapse risk by reduction of the GvL effect (3, 4). To boost the GvL effect, unmodified donor lymphocyte infusions (DLI) can be administered after alloSCT (5). A third of the patients develops clinically relevant GvHD after DLI (6). Although GvHD is a complication, it does not necessarily mean treatment failure: if GvHD resolves, the patient is unlikely to experience an eventual relapse due to the established concomitant GvL effect (7, 8). The long-term health status of patients with resolved GvHD is comparable to those who did not develop GvHD (9). Thus, GvHD is a temporary undesired state in contrast to relapse or death as definitive failures. This is captured by the endpoint current GvHD-relapse-free survival (cGRFS) which incorporates recovery from GvHD (10). Estimation of cGRFS requires advanced statistical methods that can take the end date of GvHD into account, such as multi-state models (10–12).

Different DLI strategies can be applied to achieve an optimal balance between GvL and GvHD (13). A reactive strategy is to give only therapeutic DLI to relapsed patients who need a strong alloimmune response to survive. A pre-emptive strategy administers DLI to patients based on biomarkers that may herald relapse such as mixed chimerism (MC) or minimal residual disease (MRD). In a prophylactic strategy, DLIs are given to all patients without any GvHD independent on additional biomarkers. Several factors known to influence the alloreactivity of DLI are usually taken into account to determine the DLI dose (14). First, DLIs with higher T-cell doses induce more GvHD and GvL (15). Second, patients with an unrelated donor (UD) or HLA-mismatched donor have more allo-antigens that can provoke an alloimmune response and often receive a lower dose than patients with an HLA-matched related donor (RD). Third, the DLI dose is also dependent on the timing after alloSCT, since the alloreactive potential of DLI decreases over time due to changes in the host environment (16, 17). Early after transplantation, professional antigen-presenting cells (APCs) required to activate naïve T cells are still patient-derived and therefore highly capable of activating donor-derived alloreactive T cells. Tissue damage by the conditioning regimen and infections, which occur relatively frequently during the first months after alloSCT, leads to a pro-inflammatory environment that promotes activation of alloreactive T cells (18, 19). Moreover, the conditioning-induced lymphopenia stimulates the outgrowth of (alloreactive) T cells by homeostatic proliferation and promotes activation of these T cells (20, 21). Over time after alloSCT, tissue damage is repaired, patient-derived professional APCs are replaced by donor-derived APCs, lymphopenia disappears, infections become rare, and higher T-cell doses are needed to induce a sufficient GvL effect after DLI.

Despite dose adjustments based on timing and donor type, the effect of a single DLI is highly variable between patients, ranging from patients not responding at all to patients succumbing to severe GvHD. To avoid excessive toxicity in the prophylactic or pre-emptive setting, it is crucial to better understand which factors influence the efficacy and toxicity of DLI. Since development of clinically relevant GvHD represents the clearest indicator for induction of alloreactivity after DLI, we aimed to identify risk factors for GvHD after prophylactic or pre-emptive DLI following alemtuzumab-based TCD alloSCT. Focusing on conditions that promote T-cell activation, we investigated the effects of the presence of patient-derived APCs in the bone marrow (BM) as measured by the BM chimerism level at time of DLI, the presence of lymphopenia as measured by the absolute lymphocyte count (ALC) at time of DLI, and the occurrence of viral infections (i.e., *de novo* infections or reactivations) close to DLI. We also investigated the impact of potential risk factors on the course of GvHD: GvHD only requiring short-term therapeutic systemic immunosuppression (tIS), GvHD requiring long-term tIS, or lethal GvHD. To assess their clinical relevance, we transformed these effects into cGRFS probabilities.

## 2 Methods

### 2.1 Study population

This retrospective study included all adult patients with acute myeloid leukemia, acute lymphoblastic leukemia or myelodysplastic syndrome in complete morphologic remission who received an alloSCT from a 10/10 HLA-matched donor using a standard conditioning and TCD protocol (22–24) at Leiden University Medical Center (LUMC, Leiden, The Netherlands) between 2005 and 2019. Patients scheduled to receive azacitidine or daratumumab (in 1 patient with CD38 positive acute lymphoblastic leukemia) as pharmacological maintenance therapy after alloSCT were excluded. All patients signed informed consent for data collection and analysis. Data were analyzed as of July 2021.

### 2.2 Transplantation and DLI protocol

The protocols for the myeloablative and reduced-intensity conditioning regimens (MAC and RIC, respectively), TCD and GvHD prophylaxis are described in the **Supplementary Methods**. The dose of unmodified pre-emptive and prophylactic DLI was based on donor type and timing after alloSCT. DLI at 3 months after alloSCT contained low doses of 0.3x10^6^ and 0.15x10^6^ T cells/kg in case of RD and UD, respectively. DLI at 6 months after alloSCT contained 3x10^6^ and 1.5x10^6^ T cells/kg, respectively. All patients could receive pre-emptive DLI in case of MC or MRD positivity, starting from 3 months after alloSCT. Subsequent pre-emptive DLI could be given in escalating doses with at least 3 months between DLI. Since May 2010, patients who were considered to have a high risk of relapse or who received the FLAMSA regimen received prophylactic low-dose DLI at 3 months. In addition, all eligible patients without any relapse or GvHD requiring systemic treatment received prophylactic DLI at 6 months after alloSCT regardless of chimerism or MRD status. Furthermore, selected patients could receive modified T-cell products within several clinical trials.

### 2.3 Definitions of clinical events and DLI cohorts

Relapse was defined as recurrence of at least 5% blasts on cytomorphologic BM examination, at least 1% blasts in the peripheral blood or the presence of extramedullary disease. Graft failure was defined as the occurrence of >95% mixed BM chimerism in all lineages tested or refractory granulopenia (granulocyte count <0.5x10^9^/l) in the absence of relapse and ongoing myelotoxic medication. To have a clear definition of clinically relevant GvHD with exact starting and stopping dates, essential for statistical modeling, we considered administration of tIS for acute or chronic GvHD instead of the grading of GvHD. For the analyses, we only considered tIS which was given for at least 14 days or until death, or which was stopped within 1 week before death from GvHD. In the latter case, the last week before death was added to the tIS episode. If a patient stopped tIS but had to restart tIS again within 2 months due to the recurrence of GvHD, both tIS episodes were combined into one episode. cGRFS was defined as the probability of being alive without relapse and currently not using any tIS for GvHD.

To investigate the clinical outcomes after DLI, two subcohorts were defined. The low-dose 3-month DLI cohort included all patients who were scheduled to receive a prophylactic or pre-emptive low-dose DLI at 3 months after alloSCT and received it within 6 months after alloSCT without any prior relapse, tIS for GvHD or cellular intervention besides infusion of virus-specific T cells. The 6-month DLI cohort consisted of all patients who were scheduled to receive a prophylactic or pre-emptive 6-month DLI as first DLI and received it within 9 months after alloSCT without any prior relapse, tIS for GvHD or cellular intervention besides infusion of virus-specific T cells. Both subcohorts were thus independent.

### 2.4 BM chimerism, ALC and viral infections

The methods for measuring BM chimerism, ALC and viral infections are described in the **Supplementary Methods**. The BM chimerism level was used as a measurement of the presence of patient-derived APCs in the BM at time of DLI. Three chimerism categories were defined: full donor chimerism (FDC; no detectable patient material), low MC (detectable patient material but <5%), and high MC (≥5% patient material).

Lymphopenia was defined as ALC <1000x10^6^/l, the lower limit of normal in our laboratory. For patients receiving the 3-month DLI, three ALC categories were defined: ALC <500x10^6^/l, ALC between 500 and 999x10^6^/l and ALC ≥1000x10^6^/l. For patients who received the 6-month DLI as first DLI, only two categories were used, <1000 and ≥1000x10^6^/l, since most patients had ALC ≥500x10^6^/l at that time.

All viral infections (*de novo* or reactivation) confirmed by PCR that occurred within 1 week before and 8 weeks after DLI without any prior relapse, second DLI or tIS were considered.

### 2.5 Statistical analyses

Follow-up after alloSCT was quantified using the reversed Kaplan-Meier method (25). The cumulative incidence of tIS after the first DLI (DLI1) was estimated in a competing risks model starting at time of DLI1 with start of tIS as the event of interest and relapse, death and second DLI (DLI2) as competing events. The cumulative incidence of death during treatment for GvHD from start of tIS was estimated in a competing risks model starting at time of start tIS after DLI1 with death as the event of interest and relapse, stop tIS and DLI2 as competing events.

**Figure 1:**
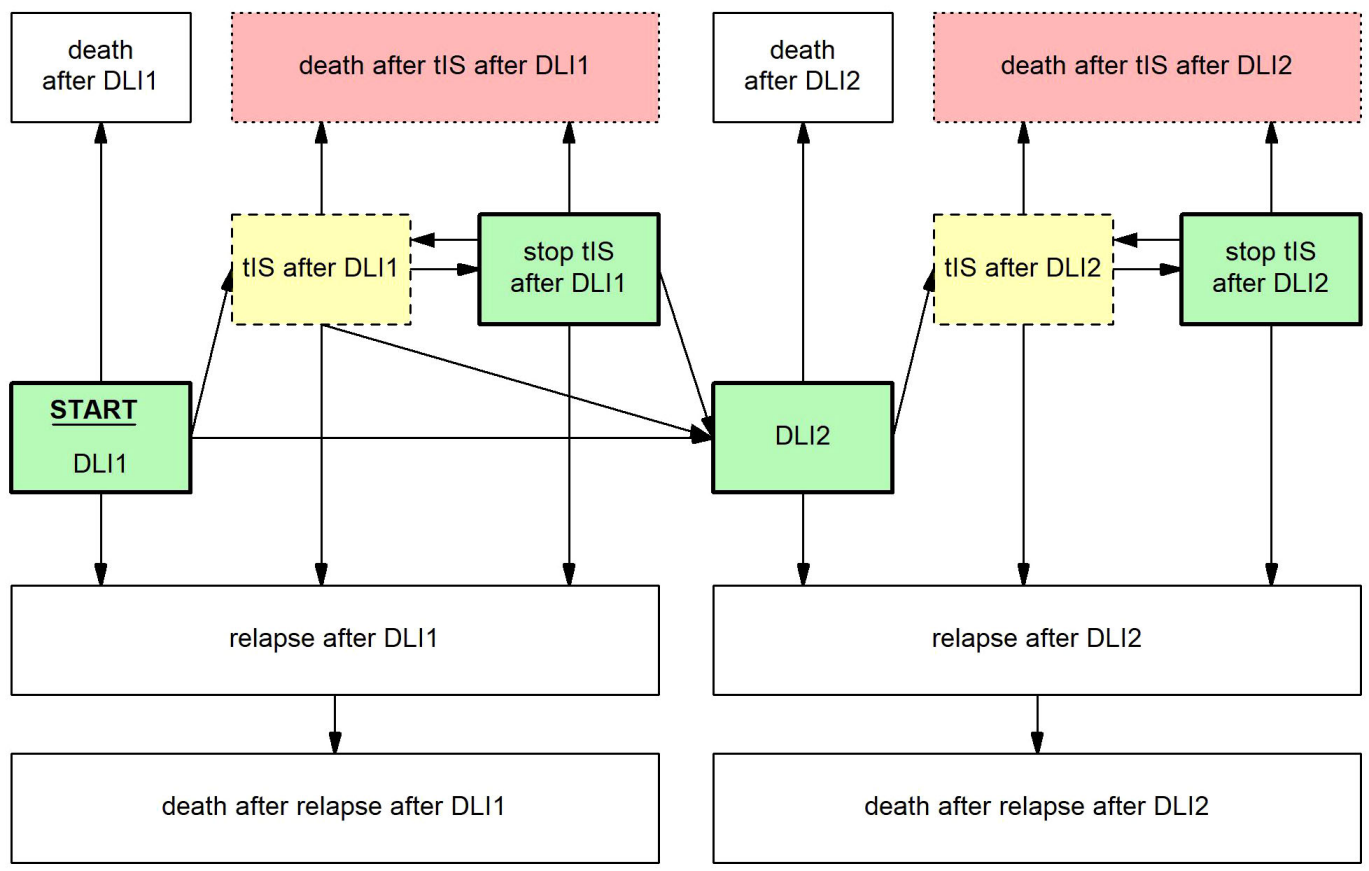
Multi-state model to evaluate the development and outcome of GvHD and other clinical events after DLI. Boxes represent states, arrows represent transitions. Starting state and time was DLI1. From here, patients could move to the state ‘relapse after DLI1’ at time of relapse, ‘death after DLI1’ at time of death, ‘tIS after DLI1’ at time of the start of tIS for GvHD and ‘DLI2’ at time of the administration of a second DLI, whichever occurred first. From the state ‘relapse after DLI1’ patients could only enter the state ‘death after relapse after DLI1’. From the state ‘tIS after DLI1’ patients could move to ‘stop tIS after DLI1’ at time of stop of all tIS, ‘relapse after DLI1’ at time of relapse, 'death after tIS after DLI1’ at time of death or ‘DLI2’ at time of the administration of a second DLI, whichever occurred first. From the state ‘stop tIS after DLI1’ patients could return to ‘tIS after DLI1’ when patients had to restart tIS for recurrent GvHD, ‘relapse after DLI1’ at time of relapse, ‘death after tIS after DLI1’ at time of death or ‘DLI2’ at time of the administration of a second DLI, whichever occurred first. After DLI2, similar states were constructed, except that any further DLIs were ignored. The cGRFS is the sum of the probabilities of all green (thick border) states, the probability of being alive with GvHD the sum of all yellow (dashed border) states, the probability of death after start of tIS for GvHD the sum of all red (dotted border) states, the RFS the sum of all green (thick border) and yellow (dashed border) states, and the OS the sum of all non-death states. For these summarizing measures, no distinction was made between states after the first DLI or after multiple DLIs.

To investigate risk factors for requiring tIS for GvHD and death during tIS and to estimate cGRFS after DLI, several Markov time-inhomogeneous multi-state models were constructed. See the **Supplementary Methods** for a brief explanation of the methodology of multi-state modelling. The structure of the main multi-state model is shown in **Figure 1**. The model used DLI1 as the starting state and time and considered the following events: death, relapse, start and stop of tIS for GvHD, and DLI2. Separate states were used for events after DLI1 and for events after second DLI (e.g., ‘relapse after DLI1’ and ‘relapse after DLI2’). The probability of cGRFS over time was calculated as the sum of the probabilities of being in one of the relevant states in the multi-state model (i.e., ‘DLI1’, ‘stop tIS after DLI1’, ‘DLI2’ and ‘stop tIS after DLI2’). The probabilities of death after start of tIS, being alive with clinically GvHD, relapse-free survival (RFS) and overall survival (OS) were calculated analogously. The outcomes after the low-dose 3-month DLI and the 6-month DLI were analyzed using two separate versions of this model, omitting all transitions and states that were not used by the included patients (**Supplementary Figures 1**, **2**).

The effects of BM chimerism, ALC and viral infections on the risk of clinically relevant GvHD after DLI were estimated using separate multivariable Cox proportional hazards regression models for the transition from ‘DLI1’ to ‘tIS after DLI1’: 3 models were fitted for the low-dose 3-month DLI and two for the 6-month DLI (only chimerism and ALC). Since donor type and conditioning/TCD regimen have been recognized as important factors for GvHD after DLI (16), conditioning/donor type (MAC UD, RIC RD and RIC UD vs MAC RD) was included in all models, while BM chimerism (low MC and high MC vs FDC), ALC (<500x10^6^/l and 500-999x10^6^/l vs ≥1000x10^6^/l for the 3-month DLI or <1000x10^6^/l vs ≥1000x10^6^/l for the 6-month DLI), or viral infection were added as the only other covariate per model. Viral infection was time-varying: patients could start as having no viral infection or as having an early viral infection if they had a viral infection during the last week before DLI. After DLI, the variable could change to ‘early viral infection’ at time of the first viral infection if this occurred within 2 weeks after DLI or to ‘late-onset viral infection’ at time of the first viral infection occurring beyond 2 weeks after DLI.

To identify risk factors for death during treatment for GvHD, univariable Cox proportional hazards regression models were fitted for the transition from ‘tIS after DLI1’ to ‘death after tIS after DLI1’ with either patient age at time of alloSCT or with the presence of early viral infection (3-month DLI) or high MC (6-month DLI). Two-sided p-values <0.05 were considered statistically significant for all Cox models. All models were based on complete cases only: patients with missing values for the included covariates were excluded.

To illustrate the impact of early viral infections on the outcome after the low-dose 3-month DLI, an extended version of the multi-state model was constructed with two starting states: ‘DLI1 without early viral infection’ for patients without any viral infection during the last week before DLI and ‘DLI1 with early viral infection’ for patients with a viral infection during this period (**Supplementary Figure 3**). To evaluate the impact of the identified transition-specific risk factors on the probability of cGRFS, the probability of being alive with GvHD, and the probability of death after start of tIS after the 6-month DLI, the Cox models for the two transitions were integrated as components in a multi-state model. This model was used to predict the outcomes after the 6-month DLI for reference patients with different baseline characteristics.

### 2.6 Software

All analyses were performed in R version 4.3.1 using the packages survival (26), prodlim (27), cmprsk (28), mstate (29), ggplot2 (30), and ComplexUpset (31).

## 3 Results

### 3.1 Cohort

388 patients were included in this study (**Supplementary Table 1**). Median follow-up after alloSCT was 76 months (interquartile range 32-110). 88 patients received the low-dose 3-month DLI prophylactically or pre-emptively at a median of 3.2 months after alloSCT (range 2.7-5.2) and 76 the 6-month DLI as first DLI at a median of 6.3 months after alloSCT (range 4.8-8.9; **Table 1**). 79 (20%) patients could not receive any DLI because of early relapse (n=44), death (n=23), or graft failure (n=12; **Supplementary Figure 4**). 66 (17%) other patients developed clinically relevant GvHD after alloSCT and therefore were not eligible for DLI. 42 patients received a modified T-cell product as part of a clinical study, and 9 received a DLI not according to our standard prophylactic/pre-emptive DLI protocol (different cell dose (n=6), DLI for a viral infection (n=2) or DLI in combination with interferon (n=1)). The remaining 28 patients did not receive any DLI within the first 9 months after alloSCT because of alloSCT before May 2010 (n=12), (temporary) donor unavailability (n=3) or physician’s decision (n=13).

**Table 1:** Baseline characteristics of the patients who received either a low-dose 3-month DLI or 6-month DLI as first DLI.

	Low-dose 3-month DLI (N = 88)	6-month DLI (N = 76)
Age at alloSCT (years)		
median (range)	58 (18–74)	57 (19-76)
Disease		
AML	59 (67%)	56 (74%)
ALL	23 (26%)	9 (12%)
MDS	6 (7%)	11 (14%)
Conditioning		
MAC: Cyclo/TBI	35 (40%)	33 (43%)
MAC: Cyclo/Bu	1 (1%)	1 (1%)
RIC: Flu/Bu*	38 (43%)	42 (55%)
RIC: Flu/Bu/Ara-C/Amsa	14 (16%)	0
Donor		
RD	39 (44%)	30 (39%)
UD	49 (56%)	46 (61%)
Graft source		
G-CSF mobilized PBSC	84 (95%)	70 (92%)
BM	4 (5%)	6 (8%)
CMV serostatus patient/donor		
+/+	43 (49%)	33 (43%)
+/-	13 (15%)	12 (16%)
-/+	6 (7%)	4 (5%)
-/-	26 (30%)	27 (36%)
EBV serostatus patient/donor		
+/+	78 (89%)	59 (78%)
+/-	6 (7%)	7 (9%)
+/unknown	0	4 (5%)
-/+	3 (3%)	6 (8%)
-/-	1 (1%)	0
Main indication of first DLI		
ALL: t(9;22)	11 (12%)	–
ALL: hypodiploidy, complex karyotype, or t(4;11)	3 (3%)	–
ALL: high white blood cell count at diagnosis	4 (5%)	–
ALL: no CR1	2 (2%)	–
AML: monosomal karyotype	10 (11%)	–
AML: complex karyotype	1 (1%)	
AML/MDS: EV1 overexpression	15 (17%)	–
AML: ASXL mutation	2 (2%)	
AML: FLT3 mutation	1 (1%)	–
AML/MDS: FLAMSA regimen	14 (16%)	
AML: progression during remission-induction	1 (1%)	–
AML/MDS: no intensive treatment or no consolidation	4 (5%)	–
AML/MDS: persisting CMML	1 (1%)	–
MRD+ at time of alloSCT	11 (12%)	–
Pre-emptive for MC	8 (9%)	34 (45%)
Standard prophylactic DLI	–	42 (55%)
BM chimerism at time of first DLI		
FDC	28 (33%)	25 (34%)
Low MC (1-4% mixed chimerism)	32 (38%)	30 (41%)
High MC (≥5% mixed chimerism)	24 (29%)	19 (26%)
Unknown	4	2
ALC at time of first DLI (x10^6^/l)		
≥1000	41 (47%)	45 (61%)
500-999	29 (33%)	20 (27%)
<500	17 (20%)	9 (12%)
Unknown	1	2

*One patient had not received a second consolidation course before transplant and received 2 days cyclophosphamide 750 mg/m2 intravenously additionally to the conditioning regimen.

Characteristics are given at time of alloSCT unless otherwise indicated.

DLI, donor lymphocyte infusion; alloSCT, allogeneic stem cell transplantation; AML, acute myeloid leukemia; ALL, acute lymphoblastic leukemia; MDS, myelodysplastic syndrome; MAC, myeloablative conditioning; RIC, reduced-intensity conditioning; Cyclo, cyclophosphamide; TBI, total body irradiation; Bu, busulfan; Flu, fludarabine; Ara-C, cytarabine; Amsa, amsacrine; RD, related donor; UD, unrelated donor; G-CSF, granulocyte-colony stimulation factor; PBSC, peripheral blood stem cells; BM, bone marrow; CMV, cytomegalovirus; EBV, Epstein-Barr virus; CR, complete morphological remission; CMML, chronic myelomonocytic leukemia; MRD, minimal residual disease; MC, mixed chimerism; FDC, full donor chimerism; ALC, absolute lymphocyte count.

### 3.2 Similar incidences of GvHD after low-dose 3-month DLI and 6-month DLI

**Figure 2:**
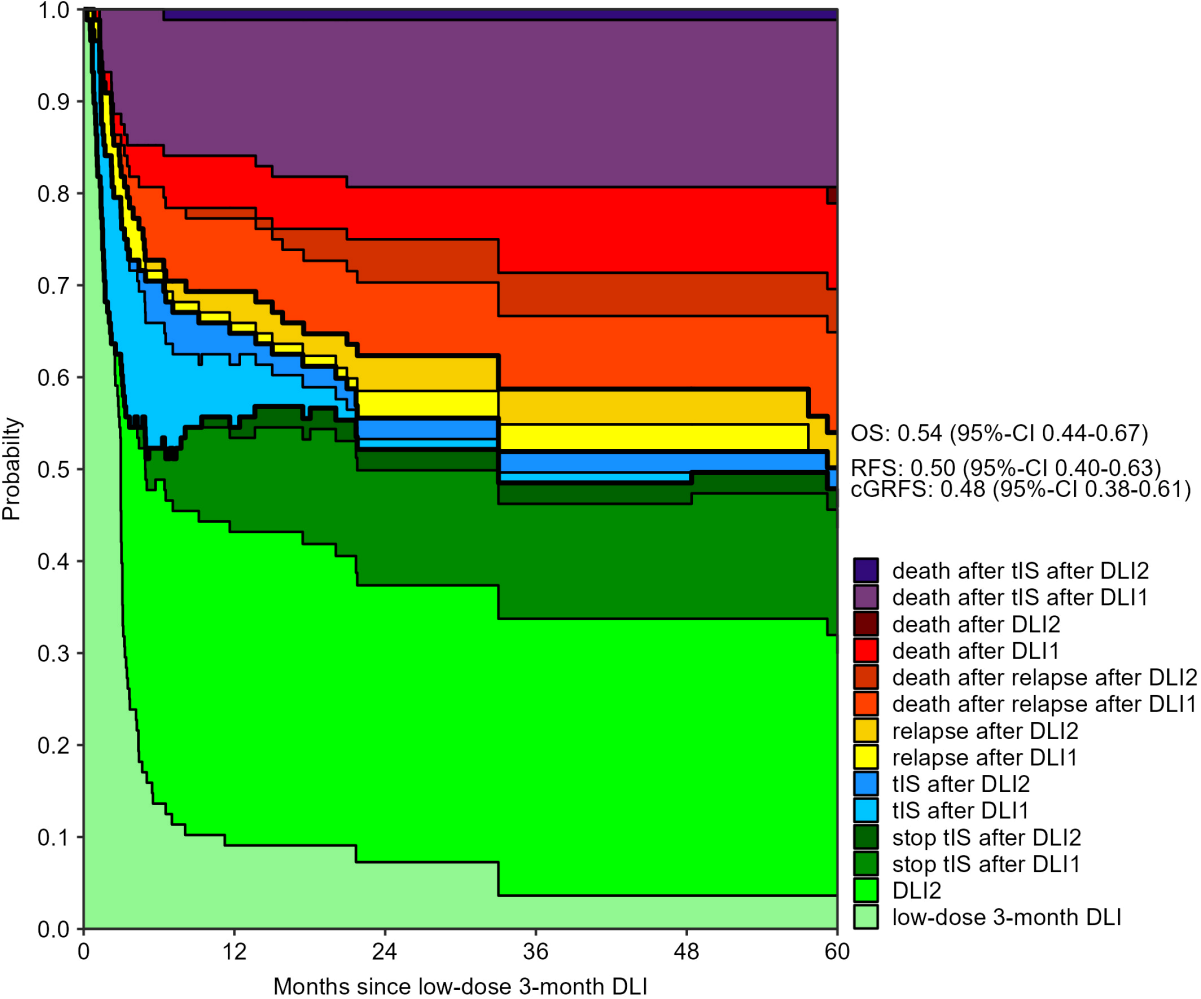
Outcomes after low-dose 3-month DLI: stacked transition probabilities from state DLI1 (low-dose 3-month DLI) estimated in the non-parametric model in **Supplementary Figure 1**. The difference between two adjacent curves represents the probability of being in the corresponding state. 39 patients reached the second DLI as planned. Bold lines show the overall survival (OS), relapse-free survival (RFS) and current GvHD-relapse-free survival (cGRFS), of which the 5-year probabilities with 95%-CI are stated next to the figure.

**Figure 3:**
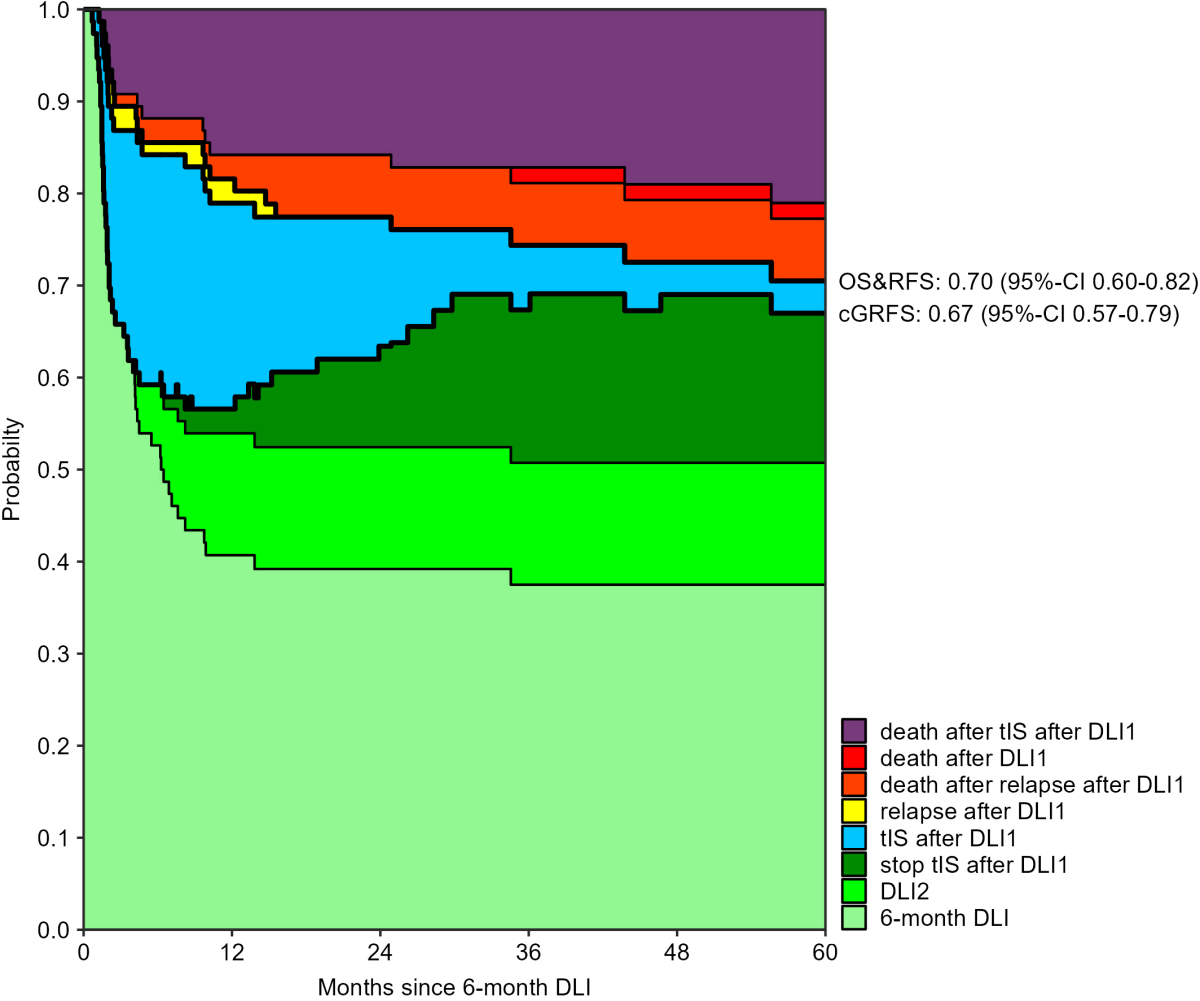
Outcomes after 6-month DLI: stacked transition probabilities from state DLI1 (6-month DLI) estimated in the non-parametric model in **Supplementary Figure 2**. The difference between two adjacent curves represents the probability of being in the corresponding state. Nine patients required a second DLI because of MC. The legend only shows the states which were occupied within 5 years after the 6-month DLI. Bold lines show the overall survival (OS), relapse-free survival (RFS) and current GvHD-relapse-free survival (cGRFS), of which the 5-year probabilities with 95%-CI are stated next to the figure.

The 3-month cumulative incidence of clinically relevant GvHD was 28% (95%-CI 20-40) after the low-dose 3-month DLI and 30% (95%-CI 22-43) after the 6-month DLI. The probability of death during tIS after one DLI was 15% (95%-CI 9-24) and 16% (95%-CI 9-27) at 12 months after the 3- and 6-month DLI, respectively (**Supplementary Figures 5**, **6**). **Figures 2**, **3** show how the state probabilities add up to the overall survival, relapse-free survival, and cGRFS probabilities. For example, the cGRFS decreased during the first months after DLI but later increased as patients with GvHD could stop their tIS after the GvHD was resolved. Notably, none of the patients with GvHD after DLI relapsed, demonstrating the concomitant GvL effect. 1- and 5-year cGRFS probabilities were 55% (95%-CI 45-66) and 48% (95%-CI 38-61) after 3-month DLI and 57% (95%-CI 46-69) and 67% (95%-CI 57-79) after 6-month DLI, respectively. Together, these data show that the tenfold dose difference effectively equalized the GvHD risk between low-dose 3-month DLI and 6-month DLI. Because 16% of patients died within 1 year after DLI during treatment for GvHD (**Figures 2**, **3**), we investigated risk factors for the development of clinically relevant GvHD and the occurrence of death during tIS.

### 3.3 Viral infections close to low-dose 3-month DLI increase the risk of GvHD after this DLI

**Figure 4:**
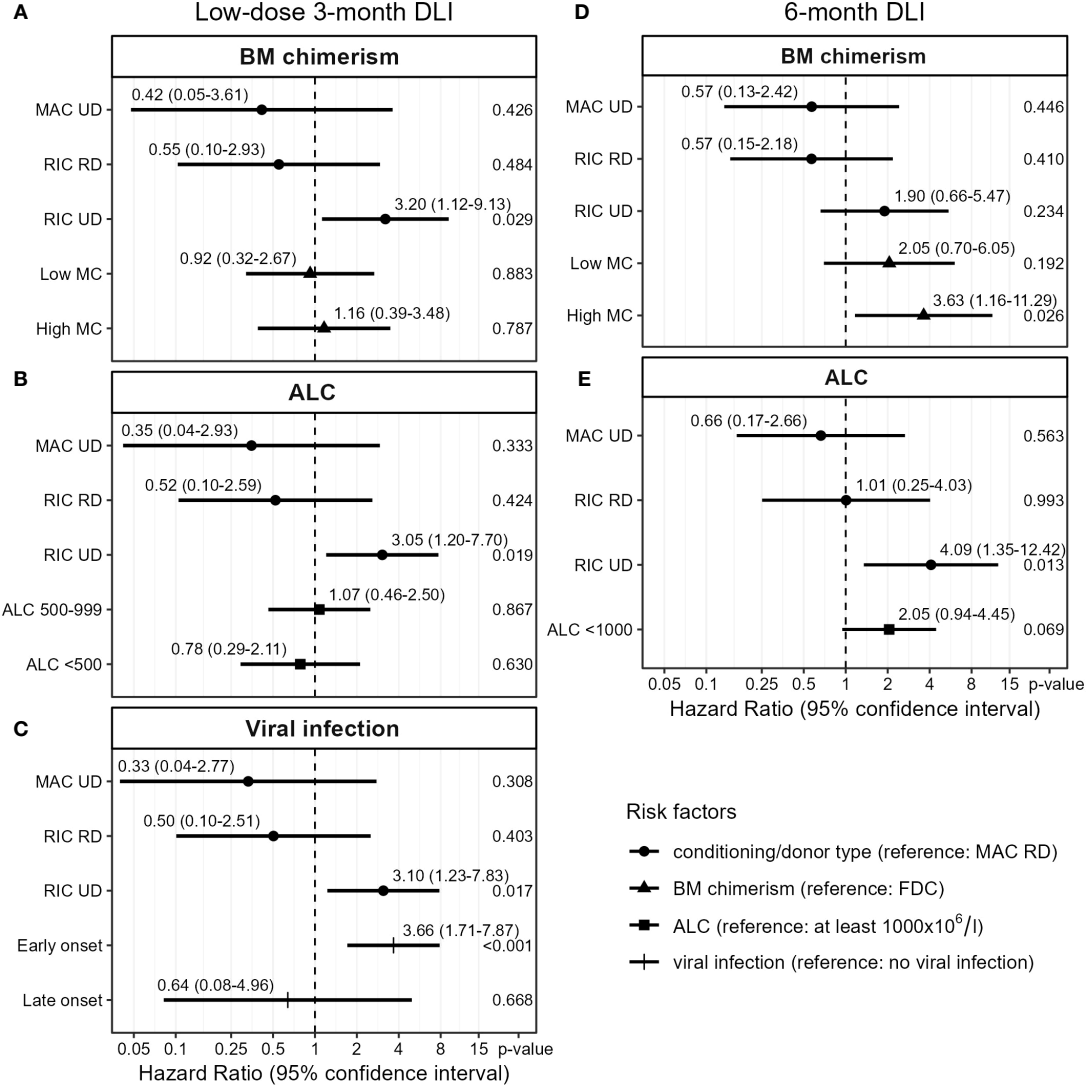
Cox proportional hazards models for the transition from first DLI to requiring tIS for GvHD (see **Figure 1**). Based on complete case analysis (**A**: n=84, **B**: n=87, **C**: n=88, **D, E**: n=74). Viral infection was treated as a time-varying covariate. DLI, donor lymphocyte infusion; BM, bone marrow; MAC, myeloablative conditioning; RIC, reduced-intensity conditioning; UD, unrelated donor; RD, related donor; low MC, 1-4% mixed chimerism; high MC, ≥5% mixed chimerism; FDC, full donor chimerism (no patient material detectable); ALC, absolute lymphocyte count (x10^6^/l).

First, we analyzed the low-dose 3-month DLI. To investigate whether the presence of patient-derived APCs in the BM increased the risk of GvHD after this DLI, we examined the chimerism model (**Figure 4A**). RIC patients with an UD had a hazard ratio (HR) of 3.2 (95%-CI 1.1-9.1) for developing GvHD compared to MAC RD patients. However, there was no significant effect of chimerism (p-values 0.9 and 0.8 for low and high MC compared to FDC, respectively) on the risk of clinically relevant GvHD after this DLI. To investigate whether lymphopenia increased the risk of GvHD after the 3-month DLI, we examined the ALC model (**Figure 4B**). Again, RIC UD was a significant risk factor while ALC showed no significant effect on GvHD after DLI (p-values 0.9 and 0.6 for ALC 500-999x10^6^/l and <500x10^6^/l compared to ≥1000x10^6^/l, respectively). We then investigated the correlation between viral infections close to the 3-month DLI and the development of GvHD after DLI. 34 of the 88 patients with a 3-month DLI had a viral infection within the last week before and first 8 weeks after DLI: 28 had an early viral infection (25 before or at time of DLI and 3 within 2 weeks after DLI) and 6 a late-onset viral infection (>2 weeks after DLI). Most common pathogens were cytomegalovirus (CMV; n=15), adenovirus (n=7) and Epstein-Barr virus (EBV; n=5; **Supplementary Figure 7A**). The model with viral infection revealed that patients with an early viral infection had a HR of 3.7 (95%-CI 1.7-7.9) for developing clinically relevant GvHD compared to those without any viral infection (**Figure 4C**). Patients with a late-onset viral infection did not have a higher risk of GvHD (p-value 0.7).

Since the ALC at time of the low-dose 3-month DLI was higher in patients with a viral infection (**Supplementary Figure 8**), viral infections may have confounded the correlation between ALC and GvHD. Therefore, to explore whether ALC is a risk factor for GvHD in the absence of viral infections, we compared the cumulative incidences of tIS for GvHD between ALC <1000x10^6^/l and ≥1000x10^6^/l in the 63 patients without any viral infection during the last week before the 3-month DLI. As we did not observe a significant difference (**Supplementary Figure 9**), there was no clear indication that viral infection acted as confounding factor. Together, these data show that viral infections close to the low-dose 3-month DLI increased the alloreactivity of this DLI leading to significantly more clinically relevant GvHD.

### 3.4 Mixed BM chimerism and lymphopenia increase the risk of GvHD after the 6-month DLI

We then investigated which risk factors were associated with the alloreactivity of the 6-month DLI. Viral infections were uncommon at the time of this DLI: of the 76 patients receiving this DLI, only 11 had a viral infection (3 early and 8 late-onset), most often EBV (n=3; **Supplementary Figure 7B**). The presence of high MC in the BM at time of DLI was a strong predictor for GvHD with a HR of 3.6 (95%-CI 1.2-11.3) compared to FDC, while patients with low MC had a nonsignificant higher risk of GvHD (HR 2.1, 95%-CI 0.7-6.1, p-value 0.19, **Figure 4D**). In the ALC model (**Figure 4E**), RIC UD was a significant risk factor for GvHD (HR 4.1, 95%-CI 1.3-12.4 compared to MAC RD). Additionally, a trend was observed for higher GvHD risk in lymphopenic patients compared to ALC ≥1000x10^6^/l (HR 2.1, 95%-CI 0.9-4.5, p-value 0.07). Together, these data show for both the low-dose 3-month DLI and the 6-month DLI, with 50% dose reduction in case of an UD, comparable risks of GvHD between patients with RD and UD after MAC but not RIC. The data indicate that mixed BM chimerism increased the risk of clinically relevant GvHD after the 6-month DLI, and suggest a similar effect of lymphopenia.

### 3.5 Risk factors for death during treatment for GvHD after DLI

To identify risk factors for death during tIS for GvHD (**Supplementary Figure 10**), we first investigated the effect of patient age. As expected, older patients seemed to have a higher risk of dying from severe GvHD after the 6-month DLI (HR 2.1 per decade, 95%-CI 0.9-5.1, p-value 0.10). Remarkably, we did not observe this association after the low-dose 3-month DLI (p-value 0.7).

Next we investigated whether the main risk factors for clinically relevant GvHD also correlated with the risk of death among those who required treatment for GvHD. For the low-dose 3-month DLI, we considered the presence of an early viral infection. We observed a nonsignificant increase in the risk of dying during tIS for GvHD for patients with an early viral infection compared to those without an early viral infection close to DLI (HR 1.8, 95%-CI 0.6-5.6, p-value 0.28, **Supplementary Figure 11**). For the 6-month DLI we considered the presence of high mixed BM chimerism at time of DLI. Patients with high MC had a nonsignificant higher risk of death during tIS for GvHD compared to those with GvHD who had FDC or low MC at time of DLI (HR 2.0, 95%-CI 0.6-6.4, p-value 0.23, **Supplementary Figure 12**). In conclusion, among those who required tIS for GvHD, older patients had a higher risk of dying during treatment after the 6-month but not the low-dose 3-month DLI. We did not observe significant associations between the risk of death during tIS and BM chimerism or viral infections. However, only one of the 53 patients with FDC at time of the low-dose 3-month DLI or 6-month DLI developed lethal GvHD.

### 3.6 Impact of early viral infection and mixed BM chimerism on the cGRFS after the low-dose 3-month DLI and 6-month DLI

**Figure 5:**
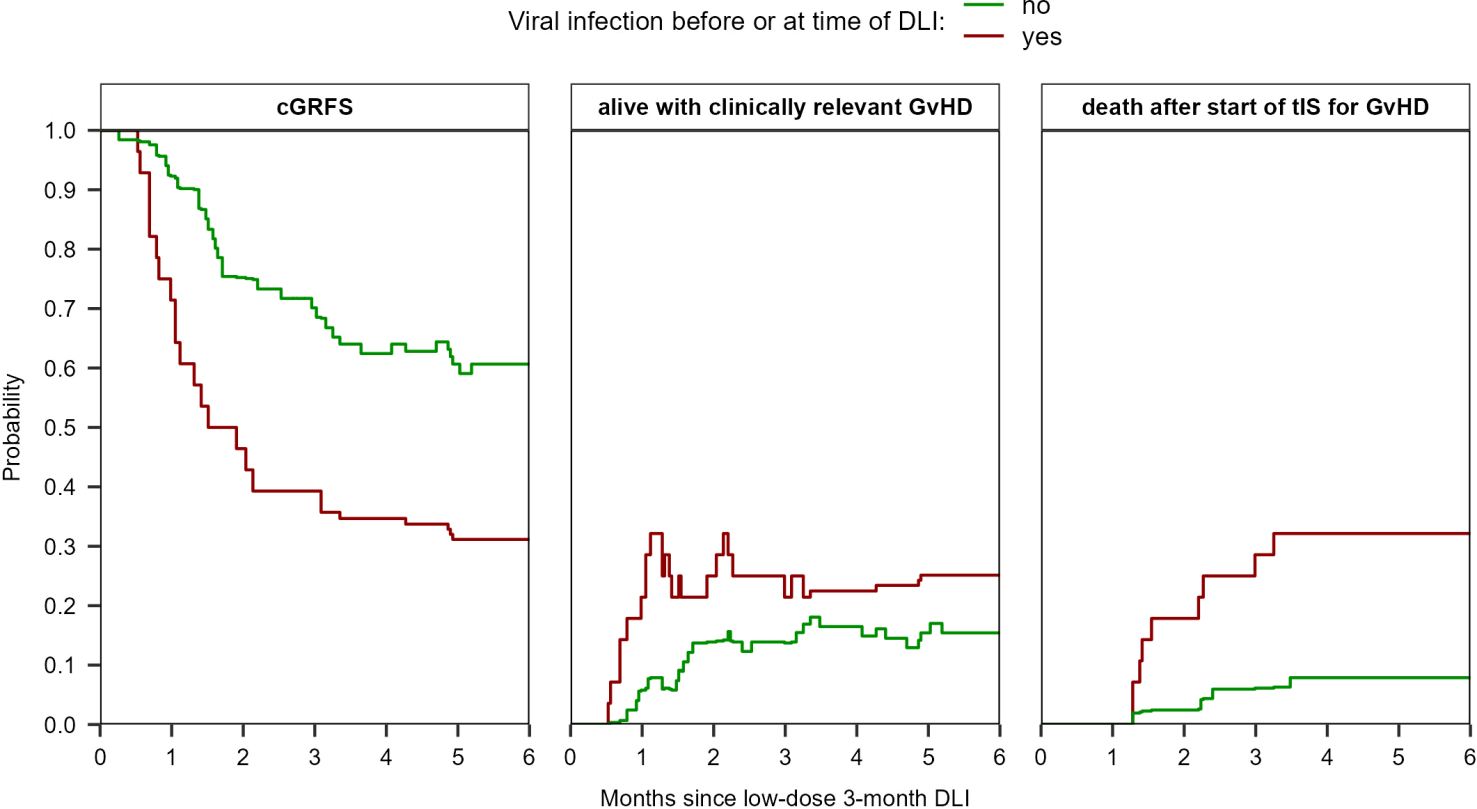
Estimated probabilities of cGRFS, being alive with clinically relevant GvHD, and of death after start of tIS for GvHD after the low-dose 3-month DLI based on the viral status at time of DLI (viral infection during the last week before DLI (n=25) or no viral infection until DLI (n=63)). The estimates are based on the non-parametric multi-state model in **Supplementary Figure 3** which has two starting states (‘DLI1 without early viral infection’ and ‘DLI1 with early viral infection’). See **Supplementary Figure 13** for the probabilities of all states separately.

The probability of having clinically relevant GvHD at 6 months after the 3-month DLI was 15% (95%-CI 9-26) for the patients without any viral infection during the last week before DLI compared to 25% (95%-CI 14-46) for the patients with a viral infection (**Figure 5**). The probability of death after start of tIS was 8% (95%-CI 4-17) compared to 32% (95%-CI 19-55), respectively. The cGRFS was 61% (95%-CI 50-73) and 31% (95%-CI 19-52), respectively.

**Figure 6:**
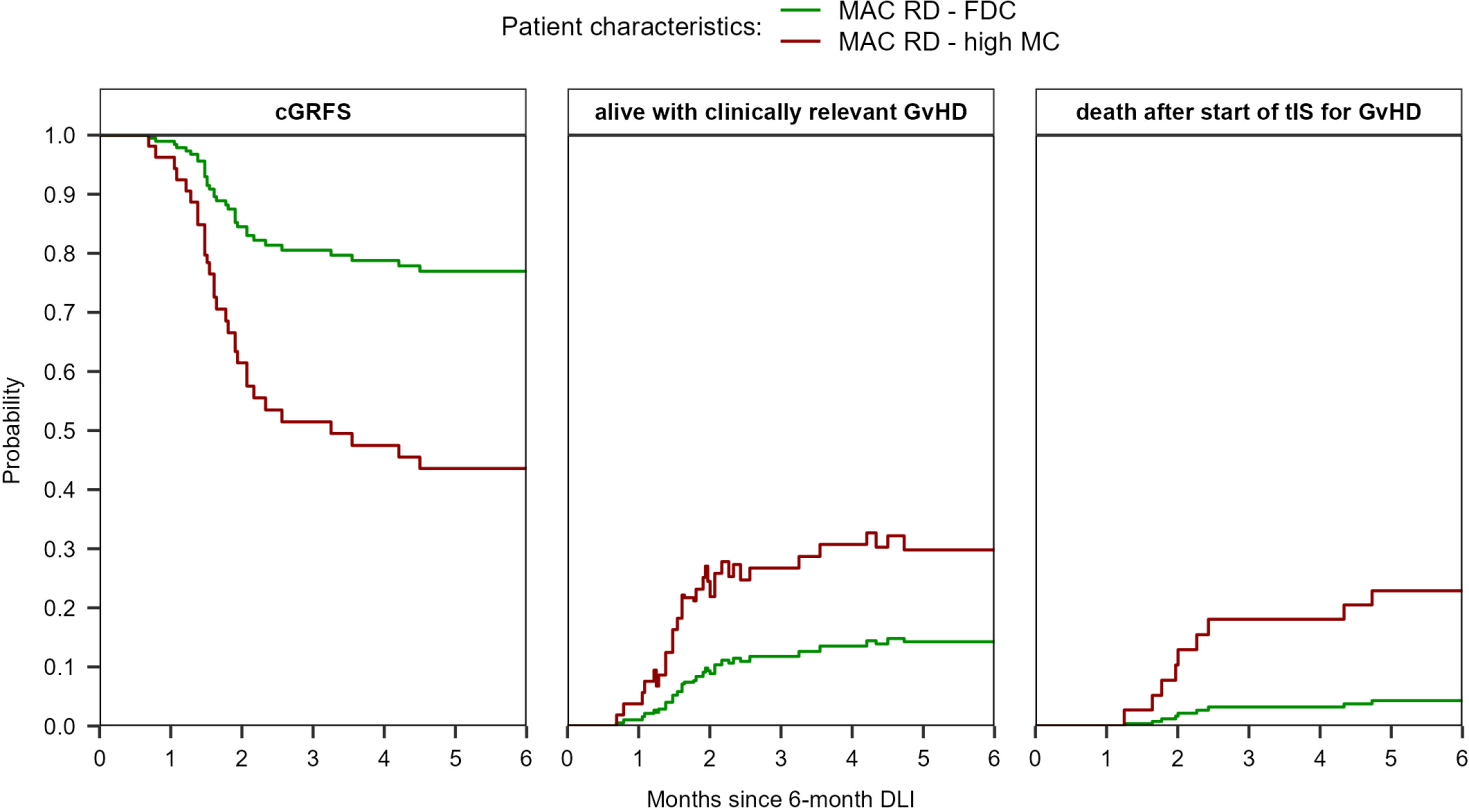
Prediction of cGRFS, being alive with clinically relevant GvHD, and of death after start of tIS for GvHD after the 6-month DLI for reference patients with different characteristics. The prediction is based on the multi-state model in **Supplementary Figure 2** with semi-parametric transition-specific proportional hazards models with BM chimerism and conditioning/donor combination as covariates for the transition from ‘DLI1’ to ‘tIS for GvHD after DLI1’ and BM chimerism (high MC vs other) for the transition from ‘tIS for GvHD after DLI1’ to ‘death after tIS after DLI1’. No covariates were assessed for the other transitions of the model.

For a MAC patient receiving a 6-month DLI from a RD, the predicted probability of having clinically relevant GvHD at 6 months after DLI was 14% (95%-CI 5-44) if the patient had FDC compared to 30% (95%-CI 11-80) if the patient had high MC, respectively (**Figure 6**). The probability of death after start of tIS was 4% (95%-CI 1-16) and 23% (95%-CI 9-58), respectively. The cGRFS for these reference patients was 77% (95%-CI 60-98) and 44% (95%-CI 19-100) at 6 months after DLI, respectively.

## 4 Discussion

In this retrospective study we investigated the outcomes after prophylactic and pre-emptive DLI following alemtuzumab-based TCD alloSCT. The tenfold dose difference between the 3- and 6-month DLI resulted in comparable risks of GvHD. For both DLIs, the 50% dose reduction in case of an UD sufficed for patients with MAC but not RIC. We demonstrate that the risk factors for GvHD after DLI depend on the setting of the DLI: at time of the 3-month DLI, the occurrence of viral infections played a major role, while for the 6-month DLI the presence of high MC in the BM was an important risk factor. The strong impact of both factors on cGRFS underlines the clinical relevance of these findings. Additionally, we observed trends for higher GvHD risk in patients with low MC or lymphopenia at time of the 6-month DLI. The very low risk of lethal GvHD for patients with FDC at time of either DLI provides further evidence for the important role of patient-derived APCs and demonstrates the safety of DLI in these patients, consistent with the matched-pair analysis by Schmid et al. (32).

Viral infection and the concomitant antiviral immune response lead to tissue damage and upregulation of HLA class II expression by non-hematopoietic cells, and induce a pro-inflammatory environment promoting activation of professional APCs and immune cells. Miller et al. showed that the occurrence of any infection (bacterial, viral or fungal) increased the risk of acute GvHD after alloSCT (33). We only considered viral infections, since these were most common in the relevant time period and most of the patients with a bacterial or fungal infection had a viral infection at the same time (data not shown). Other studies have reported associations specifically between CMV and GvHD (19, 34, 35). Previously, we demonstrated activation of alloreactive HLA-DP1-specific CD4+ T cells leading to GvHD in two patients with a CMV reactivation after a CD4+ T-cell infusion from an HLA-DP1 mismatched donor (19). Since about 80% of the patients with a 10/10 HLA-matched unrelated donor are HLA-DP mismatched (36, 37) and CMV was the most common pathogen, this mechanism could play a role in our cohort. Due to the limited number of events, we could not differentiate between the different viral pathogens.

While the role of patient-derived professional APCs in the induction of alloreactivity has been clearly demonstrated in mice (38–41), results of human studies are conflicting (42–48). This may be due to the cell subsets used for the chimerism measurement, possible bias by overrepresentation of patients with multiple DLIs, and the clinical setting. For example, Bar et al. (48) did not observe a significant correlation between BM chimerism and GvHD (HR 1.26, p-value 0.46). They however analyzed therapeutic DLI in patients who often received disease-specific treatment or cytoreduction before DLI, which most likely resulted in a more pro-inflammatory environment at time of DLI. Under these circumstances, non-hematopoietic tissues from the patient express HLA class II molecules and can act as APCs to activate donor-derived alloreactive T cells (49, 50). The presence of a pro-inflammatory environment may also be an explanation for the absent association between BM chimerism and GvHD after the 3-month DLI, as tissue damage from the conditioning and recent viral infections may still be present. Another explanation may lie in the persistence of professional patient-derived APCs in the peripheral tissues at that time. The replacement of these APCs lags behind the donor-derived BM repopulation, as long as GvHD and severe inflammation as caused by myeloablative conditioning are absent (51–53).

The relation between lymphopenia and alloreactivity of DLI has mostly been investigated in relapsed patients who often received (lymphodepleting) chemotherapy before DLI (44, 48, 54). In this context, the effects of tissue damage and APC activation interfere with estimating the effect of the lymphopenia itself on the risk of GvHD. In our setting, patients received their DLI in the absence of relapse, tissue damage and chemotherapy. Here, we observed a trend for higher GvHD risk in lymphopenic patients at time of the 6-month DLI, but not at time of the 3-month DLI.

Multi-state modeling allowed us to not only estimate the effects of risk factors on the development of GvHD and death during treatment, but also assess the impact of these factors on the probabilities of different outcomes after DLI while taking into account the hazards of all clinical events. This is a major advantage compared to less advanced statistical methods since these probabilities are more relevant for patients than HRs. Multi-state models can capture recovery after GvHD and thereby model the current GvHD burden over time, which makes cGRFS a better estimate of treatment success than GvHD-relapse-free survival (11, 12). In 2016, we introduced the endpoint treatment success, which equals cGRFS (10). During the last years, cGRFS and current immunosuppression-relapse-free survival have become more popular as outcome measures (11, 12, 55–58). However, to our knowledge, we are the first who have applied semi-parametric multi-state modeling in this context. For this, detailed data collection regarding posttransplant events and interventions as performed in this study is essential.

Our observations may eventually lead to refinement of the DLI strategy. In the prophylactic or pre-emptive setting, there is room to lower the initial DLI dose, delay the DLI or start immunosuppressive treatment on early signs of GvHD based on the anticipated risk of severe GvHD. Before implementation, our results should be validated in other clinical settings, since BM chimerism, ALC and viral infections all depend on the conditioning, donor and use or method of TCD (59–61). Larger cohorts with more events will allow for more precise prediction of alloimmune responses after DLI, not only GvHD but also the prevention of relapse. Especially the effect of BM chimerism on the risk of relapse should be investigated to confirm the correlation between MC and alloreactivity after DLI. If this is the case, the presence of MC at time of DLI can be considered for the determination of the dose of prophylactic or pre-emptive DLI.

## Data availability statement

The raw data supporting the conclusions of this article will be made available by the authors, without undue reservation.

## Ethics statement

The studies involving humans were approved by Medical Ethical Committee of Leiden University Medical Center. The studies were conducted in accordance with the local legislation and institutional requirements. The participants provided their written informed consent to participate in this study.

## Author contributions

EK: Conceptualization, Formal analysis, Investigation, Methodology, Writing – original draft, Data curation, Software, Visualization. PdB: Writing – review & editing, Resources. PB: Writing – review & editing, Resources. EM: Writing – review & editing, Resources. JT: Writing – review & editing, Resources. TS: Writing – review & editing, Resources. DL: Writing – review & editing, Resources. HV: Writing – review & editing, Resources. JF: Conceptualization, Supervision, Writing – review & editing, Methodology. CH: Conceptualization, Supervision, Writing – review & editing, Methodology. LW: Conceptualization, Supervision, Writing – review & editing, Methodology, Software.

## References

[1] Horowitz M Gale R Sondel P Goldman J Kersey J Kolb H. Graft-versus-leukemia reactions after bone marrow transplantation. Blood. (1990) 75:555–62. doi: 10.1182/blood.V75.3.555.555 2297567

[2] Falkenburg JH Jedema I. Allo-reactive T cells for the treatment of hematological Malignancies. Mol Oncol. (2015) 9:1894–903. doi: 10.1016/j.molonc.2015.10.014 PMC5528734 26578450

[3] Saad A Lamb LS. Ex vivo T-cell depletion in allogeneic hematopoietic stem cell transplant: past, present and future. Bone Marrow Transplant. (2017) 52:1241–8. doi: 10.1038/bmt.2017.22 PMC5589981 28319073

[4] Soiffer RJ Kim HT McGuirk J Horwitz ME Johnston L Patnaik MM. Prospective, randomized, double-blind, phase III clinical trial of anti-T-lymphocyte globulin to assess impact on chronic graft-versus-host disease-free survival in patients undergoing HLA-matched unrelated myeloablative hematopoietic cell transplantation. J Clin Oncol. (2017) 35:4003–11. doi: 10.1200/jco.2017.75.8177 PMC8462523 29040031

[5] Castagna L Sarina B Bramanti S Perseghin P Mariotti J Morabito L. Donor lymphocyte infusion after allogeneic stem cell transplantation. Transfus Apher Sci. (2016) 54:345–55. doi: 10.1016/j.transci.2016.05.011 27216544

[6] Schmid C Labopin M Schaap N Veelken H Brecht A Stadler M. Long-term results and GvHD after prophylactic and preemptive donor lymphocyte infusion after allogeneic stem cell transplantation for acute leukemia. Bone Marrow Transplant. (2022) 57:215–23. doi: 10.1038/s41409-021-01515-3 PMC8821014 34750562

[7] Weisdorf D Zhang MJ Arora M Horowitz MM Rizzo JD Eapen M. Graft-versus-host disease induced graft-versus-leukemia effect: greater impact on relapse and disease-free survival after reduced intensity conditioning. Biol Blood Marrow Transplant. (2012) 18:1727–33. doi: 10.1016/j.bbmt.2012.06.014 PMC3472079 22766220

[8] Yeshurun M Weisdorf D Rowe JM Tallman MS Zhang MJ Wang HL. The impact of the graft-versus-leukemia effect on survival in acute lymphoblastic leukemia. Blood advances. (2019) 3:670–80. doi: 10.1182/bloodadvances.2018027003 PMC6391668 30808685

[9] Fraser CJ Bhatia S Ness K Carter A Francisco L Arora M. Impact of chronic graft-versus-host disease on the health status of hematopoietic cell transplantation survivors: a report from the Bone Marrow Transplant Survivor Study. Blood. (2006) 108:2867–73. doi: 10.1182/blood-2006-02-003954 PMC1895593 16788100

[10] Eefting M de Wreede LC Halkes CJ von dem Borne PA Kersting S Marijt EW. Multi-state analysis illustrates treatment success after stem cell transplantation for acute myeloid leukemia followed by donor lymphocyte infusion. Haematologica. (2016) 101:506–14. doi: 10.3324/haematol.2015.136846 PMC5004390 26802054

[11] Solomon SR Sizemore C Zhang X Ridgeway M Solh M Morris LE. Current graft-versus-host disease-free, relapse-free survival: A dynamic endpoint to better define efficacy after allogenic transplant. Biol Blood Marrow Transplant. (2017) 23:1208–14. doi: 10.1016/j.bbmt.2017.02.022 28390985

[12] Holtan SG Zhang L DeFor TE Bejanyan N Arora M Rashidi A. Dynamic graft-versus-host disease-free, relapse-free survival: multistate modeling of the morbidity and mortality of allotransplantation. Biol Blood Marrow Transplant. (2019) 25:1884–9. doi: 10.1016/j.bbmt.2019.05.015 PMC6755055 31128328

[13] Biederstädt A Rezvani K. How I treat high-risk acute myeloid leukemia using preemptive adoptive cellular immunotherapy. Blood. (2023) 141:22–38. doi: 10.1182/blood.2021012411 35512203 PMC10023741

[14] Falkenburg J Schmid C Kolb H Locatelli F Kuball J. Delayed transfer of immune cells or the art of donor lymphocyte infusion. In: Carreras E Dufour C Mohty M Kroger N, editors. The EBMT Handbook Hematopoietic Stem Cell Transplantation and Cellular Therapies. Cham: Springer, Cham (2019). p. 443–8.

[15] Mackinnon S Papadopoulos EB Carabasi MH Reich L Collins NH Boulad F. Adoptive immunotherapy evaluating escalating doses of donor leukocytes for relapse of chronic myeloid leukemia after bone marrow transplantation: separation of graft-versus-leukemia responses from graft-versus-host disease. Blood. (1995) 86:1261–8. doi: 10.1182/blood.V86.4.1261.bloodjournal8641261 7632930

[16] Yun HD Waller EK. Finding the sweet spot for donor lymphocyte infusions. Biol Blood Marrow Transplant. (2013) 19:507–8. doi: 10.1016/j.bbmt.2013.02.005 23416853

[17] Chakraverty R Flutter B Fallah-Arani F Eom HS Means T Andreola G. The host environment regulates the function of CD8+ graft-versus-host-reactive effector cells. J Immunol (Baltimore Md 1950). (2008) 181:6820–8. doi: 10.4049/jimmunol.181.10.6820 18981100

[18] Ferrara JL Levine JE Reddy P Holler E. Graft-versus-host disease. Lancet. (2009) 373:1550–61. doi: 10.1016/s0140-6736(09)60237-3 PMC2735047 19282026

[19] Stevanovic S van Bergen CA van Luxemburg-Heijs SA van der Zouwen B Jordanova ES Kruisselbrink AB. HLA class II upregulation during viral infection leads to HLA-DP-directed graft-versus-host disease after CD4+ donor lymphocyte infusion. Blood. (2013) 122:1963–73. doi: 10.1182/blood-2012-12-470872 23777765

[20] Thiant S Yakoub-Agha I Magro L Trauet J Coiteux V Jouet JP. Plasma levels of IL-7 and IL-15 in the first month after myeloablative BMT are predictive biomarkers of both acute GVHD and relapse. Bone Marrow Transplant. (2010) 45:1546–52. doi: 10.1038/bmt.2010.13 20190846

[21] Thiant S Labalette M Trauet J Coiteux V de Berranger E Dessaint JP. Plasma levels of IL-7 and IL-15 after reduced intensity conditioned allo-SCT and relationship to acute GVHD. Bone Marrow Transplant. (2011) 46(10):1374–81. doi: 10.1038/bmt.2010.300 21132028

[22] Barge RM Starrenburg CW Falkenburg JH Fibbe WE Marijt EW Willemze R. Long-term follow-up of myeloablative allogeneic stem cell transplantation using Campath "in the bag" as T-cell depletion: the Leiden experience. Bone Marrow Transplant. (2006) 37:1129–34. doi: 10.1038/sj.bmt.1705385 16757974

[23] von dem Borne PA Beaumont F Starrenburg CW Oudshoorn M Hale G Falkenburg JH. Outcomes after myeloablative unrelated donor stem cell transplantation using both in *vitro* and in *vivo* T-cell depletion with alemtuzumab. Haematologica. (2006) 91:1559–62. 17082014

[24] von dem Borne PA Starrenburg CW Halkes SJ Marijt WA Fibbe WE Falkenburg JH. Reduced-intensity conditioning allogeneic stem cell transplantation with donor T-cell depletion using alemtuzumab added to the graft ('Campath in the bag'). Curr Opin Oncol. (2009) 21:S27–9. doi: 10.1097/01.cco.0000357472.76337.0e 19561408

[25] Schemper M Smith TL. A note on quantifying follow-up in studies of failure time. Control Clin Trials. (1996) 17:343–6. doi: 10.1016/0197-2456(96)00075-x 8889347

[26] Therneau TM Grambsch PM. Modeling Survival Data: Extending the Cox Model. New York: Springer (2000).

[27] Gerds TA. prodlim: Product-Limit Estimation for Censored Event History Analysis (2019). Available at: https://cran.r-project.org/package=prodlim.

[28] Gray B. cmprsk: Subdistribution Analysis of Competing Risks (2022). Available at: https://CRAN.R-project.org/package=cmprsk.

[29] De Wreede LC Fiocco M Putter H. mstate: an R package for the analysis of competing risks and multi-state models. J Stat Software. (2011) 38:1–30. doi: 10.18637/jss.v038.i07

[30] Wickham H. ggplot2: Elegant Graphics for Data Analysis. New York: Springer-Verlag New York (2016).

[31] Krassowski M. ComplexUpset. (2020). doi: 10.5281/zenodo.3700590.

[32] Schmid C Labopin M Schaap N Veelken H Schleuning M Stadler M. Prophylactic donor lymphocyte infusion after allogeneic stem cell transplantation in acute leukaemia - a matched pair analysis by the Acute Leukaemia Working Party of EBMT. Br J Haematol. (2019) 184:782–7. doi: 10.1111/bjh.15691 30467839

[33] Miller HK Braun TM Stillwell T Harris AC Choi S Connelly J. Infectious risk after allogeneic hematopoietic cell transplantation complicated by acute graft-versus-host disease. Biol Blood Marrow Transplant. (2017) 23:522–8. doi: 10.1016/j.bbmt.2016.12.630 PMC5551893 28017733

[34] Broers AE van der Holt R van Esser JW Gratama JW Henzen-Logmans S Kuenen-Boumeester V. Increased transplant-related morbidity and mortality in CMV-seropositive patients despite highly effective prevention of CMV disease after allogeneic T-cell-depleted stem cell transplantation. Blood. (2000) 95:2240–5. doi: 10.1182/blood.V95.7.2240 10733491

[35] Cantoni N Hirsch HH Khanna N Gerull S Buser A Bucher C. Evidence for a bidirectional relationship between cytomegalovirus replication and acute graft-versus-host disease. Biol Blood Marrow Transplant. (2010) 16:1309–14. doi: 10.1016/j.bbmt.2010.03.020 20353832

[36] Shaw BE Gooley TA Malkki M Madrigal JA Begovich AB Horowitz MM. The importance of HLA-DPB1 in unrelated donor hematopoietic cell transplantation. Blood. (2007) 110:4560–6. doi: 10.1182/blood-2007-06-095265 17726164

[37] Fleischhauer K Shaw BE Gooley T Malkki M Bardy P Bignon JD. Effect of T-cell-epitope matching at HLA-DPB1 in recipients of unrelated-donor haemopoietic-cell transplantation: a retrospective study. Lancet Oncol. (2012) 13:366–74. doi: 10.1016/s1470-2045(12)70004-9 PMC3813000 22340965

[38] Shlomchik WD Couzens MS Tang CB McNiff J Robert ME Liu J. Prevention of graft versus host disease by inactivation of host antigen-presenting cells. Sci (New York NY). (1999) 285:412–5. doi: 10.1126/science.285.5426.412 10411505

[39] Mapara MY Kim YM Wang SP Bronson R Sachs DH Sykes M. Donor lymphocyte infusions mediate superior graft-versus-leukemia effects in mixed compared to fully allogeneic chimeras: a critical role for host antigen-presenting cells. Blood. (2002) 100:1903–9. doi: 10.1182/blood-2002-01-0023 12176915

[40] Merad M Hoffmann P Ranheim E Slaymaker S Manz MG Lira SA. Depletion of host Langerhans cells before transplantation of donor alloreactive T cells prevents skin graft-versus-host disease. Nat Med. (2004) 10:510–7. doi: 10.1038/nm1038 PMC4727841 15098028

[41] Xia G Truitt RL Johnson BD. Graft-versus-leukemia and graft-versus-host reactions after donor lymphocyte infusion are initiated by host-type antigen-presenting cells and regulated by regulatory T cells in early and long-term chimeras. Biol Blood Marrow Transplant. (2006) 12:397–407. doi: 10.1016/j.bbmt.2005.11.519 16545723

[42] Levenga H Woestenenk R Schattenberg AV Maas F Jansen JH Raymakers R. Dynamics in chimerism of T cells and dendritic cells in relapsed CML patients and the influence on the induction of alloreactivity following donor lymphocyte infusion. Bone Marrow Transplant. (2007) 40:585–92. doi: 10.1038/sj.bmt.1705777 17637687

[43] Peggs KS Thomson K Hart DP Geary J Morris EC Yong K. Dose-escalated donor lymphocyte infusions following reduced intensity transplantation: toxicity, chimerism, and disease responses. Blood. (2004) 103:1548–56. doi: 10.1182/blood-2003-05-1513 14576063

[44] Shaw BE Byrne JL Das-Gupta E Carter GI Russell NH. The impact of chimerism patterns and predonor leukocyte infusion lymphopenia on survival following T cell-depleted reduced intensity conditioned transplants. Biol Blood Marrow Transplant. (2007) 13:550–9. doi: 10.1016/j.bbmt.2006.12.451 17448914

[45] Lutz C Massenkeil G Nagy M Neuburger S Tamm I Rosen O. A pilot study of prophylactic donor lymphocyte infusions to prevent relapse in adult acute lymphoblastic leukemias after allogeneic hematopoietic stem cell transplantation. Bone Marrow Transplant. (2008) 41:805–12. doi: 10.1038/sj.bmt.1705981 18195682

[46] Liga M Triantafyllou E Tiniakou M Lambropoulou P Karakantza M Zoumbos NC. High alloreactivity of low-dose prophylactic donor lymphocyte infusion in patients with acute leukemia undergoing allogeneic hematopoietic cell transplantation with an alemtuzumab-containing conditioning regimen. Biol Blood Marrow Transplant. (2013) 19:75–81. doi: 10.1016/j.bbmt.2012.07.021 22871557

[47] Caldemeyer LE Akard LP Edwards JR Tandra A Wagenknecht DR Dugan MJ. Donor Lymphocyte Infusions Used to Treat Mixed-Chimeric and High-Risk Patient Populations in the Relapsed and Nonrelapsed Settings after Allogeneic Transplantation for Hematologic Malignancies Are Associated with High Five-Year Survival if Persistent Full Donor Chimerism Is Obtained or Maintained. Biol Blood Marrow Transplant. (2017) 23:1989–97. doi: 10.1016/j.bbmt.2017.07.007 28712934

[48] Bar M Sandmaier BM Inamoto Y Bruno B Hari P Chauncey T. Donor lymphocyte infusion for relapsed hematological Malignancies after allogeneic hematopoietic cell transplantation: prognostic relevance of the initial CD3+ T cell dose. Biol Blood Marrow Transplant. (2013) 19:949–57. doi: 10.1016/j.bbmt.2013.03.001 PMC3846289 23523892

[49] Collins T Korman AJ Wake CT Boss JM Kappes DJ Fiers W. Immune interferon activates multiple class II major histocompatibility complex genes and the associated invariant chain gene in human endothelial cells and dermal fibroblasts. Proc Natl Acad Sci United States America. (1984) 81:4917–21. doi: 10.1073/pnas.81.15.4917 PMC391603 6431411

[50] Koyama M Kuns RD Olver SD Raffelt NC Wilson YA Don AL. Recipient nonhematopoietic antigen-presenting cells are sufficient to induce lethal acute graft-versus-host disease. Nat Med. (2011) 18:135–42. doi: 10.1038/nm.2597 22127134

[51] Collin MP Hart DN Jackson GH Cook G Cavet J Mackinnon S. The fate of human Langerhans cells in hematopoietic stem cell transplantation. J Exp Med. (2006) 203:27–33. doi: 10.1084/jem.20051787 16390938 PMC2118090

[52] Mielcarek M Kirkorian AY Hackman RC Price J Storer BE Wood BL. Langerhans cell homeostasis and turnover after nonmyeloablative and myeloablative allogeneic hematopoietic cell transplantation. Transplantation. (2014) 98:563–8. doi: 10.1097/tp.0000000000000097 PMC4149838 24717220

[53] van Balen P van der Zouwen B Kruisselbrink AB Eefting M Szuhai K Jordanova ES. Tissue Damage Caused by Myeloablative, but Not Non-Myeloablative, Conditioning before Allogeneic Stem Cell Transplantation Results in Dermal Macrophage Recruitment without Active T-Cell Interaction. Front Immunol. (2018) 9:331. doi: 10.3389/fimmu.2018.00331 29535719 PMC5835032

[54] Miller JS Weisdorf DJ Burns LJ Slungaard A Wagner JE Verneris MR. Lymphodepletion followed by donor lymphocyte infusion (DLI) causes significantly more acute graft-versus-host disease than DLI alone. Blood. (2007) 110:2761–3. doi: 10.1182/blood-2007-05-090340 PMC1988949 17579184

[55] Kanakry CG Bolaños-Meade J Kasamon YL Zahurak M Durakovic N Furlong T. Low immunosuppressive burden after HLA-matched related or unrelated BMT using posttransplantation cyclophosphamide. Blood. (2017) 129:1389–93. doi: 10.1182/blood-2016-09-737825 PMC5345732 28049637

[56] Kawamura K Nakasone H Kurosawa S Yoshimura K Misaki Y Gomyo A. Refractory graft-versus-host disease-free, relapse-free survival as an accurate and easy-to-calculate endpoint to assess the long-term transplant success. Biol Blood Marrow Transplant. (2018) 24:1521–6. doi: 10.1016/j.bbmt.2018.02.004 29476953

[57] Bluhmki T Schmoor C Finke J Schumacher M Socié G Beyersmann J. Relapse- and immunosuppression-free survival after hematopoietic stem cell transplantation: how can we assess treatment success for complex time-to-event endpoints? Biol Blood Marrow Transplant. (2020) 26:992–7. doi: 10.1016/j.bbmt.2020.01.001 31927103

[58] Carnevale-Schianca F Caravelli D Gallo S Becco P Paruzzo L Poletto S. Post-transplant cyclophosphamide and tacrolimus-mycophenolate mofetil combination governs GVHD and immunosuppression need, reducing late toxicities in allogeneic peripheral blood hematopoietic cell transplantation from HLA-matched donors. J Clin Med. (2021) 10. doi: 10.3390/jcm10061173 PMC7998305 33799685

[59] Antin JH Childs R Filipovich AH Giralt S Mackinnon S Spitzer T. Establishment of complete and mixed donor chimerism after allogeneic lymphohematopoietic transplantation: recommendations from a workshop at the 2001 Tandem Meetings of the International Bone Marrow Transplant Registry and the American Society of Blood and Marrow Transplantation. Biol Blood Marrow Transplant. (2001) 7:473–85. doi: 10.1053/bbmt.2001.v7.pm11669214 11669214

[60] Ando T Tachibana T Tanaka M Suzuki T Ishiyama Y Koyama S. Impact of graft sources on immune reconstitution and survival outcomes following allogeneic stem cell transplantation. Blood advances. (2020) 4:408–19. doi: 10.1182/bloodadvances.2019001021 PMC6988395 31990335

[61] Bejanyan N Brunstein CG Cao Q Lazaryan A Luo X Curtsinger J. Delayed immune reconstitution after allogeneic transplantation increases the risks of mortality and chronic GVHD. Blood advances. (2018) 2:909–22. doi: 10.1182/bloodadvances.2017014464 PMC5916001 29678809

