# Supplementary material for: Risk factors for graft-versus-host-disease after donor lymphocyte infusion following T-cell depleted allogeneic stem cell transplantation
Source: Front Immunol. 2024 Mar 13;15:1335341. doi: 10.3389/fimmu.2024.1335341 (PMC10966113; doi:10.3389/fimmu.2024.1335341)
Supplement: Supplementary file 1 [file DataSheet_1.pdf]

Supplementary Material

1 Supplementary Methods

Transplantation protocol

Myeloablative conditioning (MAC) consisted of cyclophosphamide (2 days 60 mg/kg intravenously) combined with either 9 Gy total body irradiation or busulfan (4 days 4x0.8 mg/kg intravenously). Reduced-intensity conditioning (RIC) consisted either of fludarabine (6 days 50 mg/m^2 orally or 30 mg/m^2 intravenously) and busulfan (2 days 4x0.8 mg/kg intravenously), or the FLAMSA regimen: fludarabine (5 days 30 mg/m^2 intravenously), cytarabine (4 days 2000 mg/m^2 intravenously), amsacrine (4 days 100 mg/m^2 intravenously) and busulfan (4 days 4x0.8 mg/kg intravenously).

Standard *in-vitro* TCD was performed by adding 20 mg alemtuzumab (Sanofi Genzyme) to the graft.(1) Additional *in-vivo* TCD depended on the donor type and conditioning regimen: MAC patients with a RD did not receive any *in-vivo* TCD. All other patients received 15 mg alemtuzumab intravenously on days -6 and -5 (MAC) or on days -4 and -3 (RIC). Before June 2007, RIC patients with an UD received 10 mg/kg horse-derived anti-thymocyte globulin (Lymphoglobulin, Genzyme) additionally on day -4 until day -1. After Lymphoglobulin was withdrawn from the market, RIC patients with an UD first received no anti-thymocyte globulin (alloSCT between June 2007 and September 2009) and later received rabbit-derived anti-thymocyte globulin (Thymoglobulin, Sanofi Genzyme) additionally on day -2 (until April 2010 2mg/kg and thereafter 1mg/kg). Only MAC patients with an UD received posttransplant ciclosporin as GvHD prophylaxis, which was tapered from 1 month with the aim to stop within 3 months after alloSCT.

BM chimerism, ALC and viral infections

For the BM chimerism at time of DLI, we used the BM sample that was closest to DLI and taken within 5 weeks before and 1 week after DLI. BM chimerism was measured in total BM leukocytes by short-tandem-repeat PCR or, for patients transplanted before 2007 with a sex-mismatched donor, by FISH analysis using Vysis CEP X/Y probes. The lower detection limit of the chimerism analyses was 1-2%, depending on the method and the selected markers. For patients without any evaluable BM chimerism measurement during this period but whose last measurement before and first measurement after DLI belonged to the same chimerism category (FDC, low MC or high MC), this category was taken as the BM chimerism status at time of DLI.

ALC was calculated by the sum of the absolute numbers of circulating T cells, B cells and NK cells as measured on anticoagulated fresh venous blood by flow cytometry with bead calibration (Trucount tubes, Becton Dickinson) with a lower detection limit of 0.5x10^6 cells/l. If these counts were unavailable, the lymphocyte count by manual blood smear was used. For the ALC at time of DLI, the closest measurement within 2 weeks before and 1 week after DLI was taken. For patients without any ALC measurement during this period but whose last ALC before and first ALC after DLI belonged to the same category (<500, 500-999 or ≥1000x10^6/l for the low-dose 3-month DLI and <1000 or ≥1000x10^6/l for the 6-month DLI), this category was taken as the ALC at time of DLI.

Cytomegalovirus (CMV) and Epstein-Barr virus (EBV) were monitored weekly by PCR on peripheral blood samples in all patients. Single positive values of CMV or EBV below log 2.4 were not considered. PCRs on other viruses were only performed in symptomatic patients. For the analyses, only the first viral infection was used.

Multi-state modelling

In a multi-state model patients move between states at the occurrence of clinical events or treatments. Transitions define which routes between states are allowed (for instance the transition from the state DLI to the state GvHD).(2) In a Markov model, the hazard of making a certain transition only depends on the current state and the time since start, which is in this case the first DLI. Each transition hazard can either be estimated without taking covariates into account (non-parametrically) or can be analyzed by means of a transition-specific Cox proportional hazards model (semi-parametric approach). The baseline hazards and the hazard ratios are the building blocks for the calculation of the transition probabilities, which represent the probabilities of being in each of the states over time. For example, in a semi-parametric model the probability of being alive with GvHD depends on the baseline hazard of GvHD, the effects of risk factors for GvHD, and the risks of death and disappearance of GvHD symptoms. Confidence intervals for the probabilities of cGRFS, death after start of tIS, being alive with clinically relevant GvHD, RFS and OS were calculated based on the estimated variance-covariance matrix of all transition probabilities.

References

1. von dem Borne PA, Beaumont F, Starrenburg CW, et al. Outcomes after myeloablative unrelated donor stem cell transplantation using both in vitro and in vivo T-cell depletion with alemtuzumab. *Haematologica*. 2006;91(11):1559-62.
2. Putter H, Fiocco M, Geskus RB. Tutorial in biostatistics: competing risks and multi-state models. *Stat Med*. 2007;26(11):2389-430. doi:10.1002/sim.2712

2 Supplementary Tables

	Total cohort (all included patients with alloSCT; N = 388)
**Age at alloSCT (years)**	
median (range)	54 (18-78)
**Disease**	
acute myeloid leukemia	260 (67%)
acute lymphoblastic leukemia	85 (22%)
myelodysplastic syndrome	43 (11%)
**Conditioning**	
MAC: Cyclo/TBI	196 (51%)
MAC: Cyclo/Bu	9 (2%)
RIC: Flu/Bu*	167 (43%)
RIC: Flu/Bu/Ara-C/Amsa	16 (4%)
**Donor**	
RD	165 (43%)
UD	223 (57%)
**Graft source**	
G-CSF mobilized PBSC	368 (95%)
BM	20 (5%)
**CMV serostatus patient/donor**	
+/+	169 (44%)
+/-	70 (18%)
-/+	29 (7%)
-/-	120 (31%)
**EBV serostatus patient/donor**	
+/+	323 (83%)
+/-	30 (8%)
+/unknown	15 (4%)
-/+	18 (5%)
-/-	2 (1%)

Supplementary Table 1. Baseline characteristics of all included patients who received an alloSCT. alloSCT, allogeneic stem cell transplantation; MAC, myeloablative conditioning; RIC, reduced-intensity conditioning; Cyclo, cyclophosphamide; TBI, total body irradiation; Bu, busulfan; Flu, fludarabine; Ara-C, cytarabine; Amsa, amsacrine; RD, related donor; UD, unrelated donor; G-CSF, granulocyte-colony stimulation factor; PBSC, peripheral blood stem cells; BM, bone marrow; CMV, cytomegalovirus; EBV, Epstein-Barr virus

*One patient had not received a second consolidation course before transplant and received 2 days cyclophosphamide 750 mg/m2 intravenously additionally to the conditioning regimen.

3 Supplementary Figures

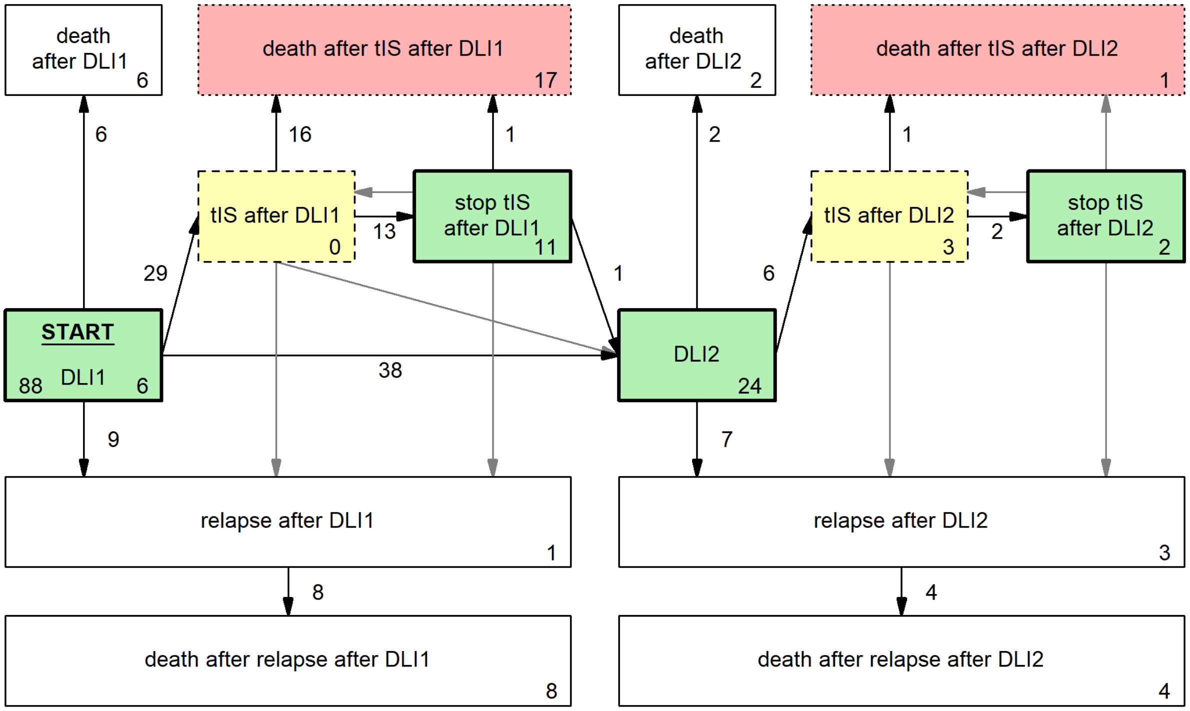

Supplementary Figure 1. Multi-state model for low-dose 3-month DLI. Boxes represent states and arrows represent the transitions between the states. Grey transitions were not used by any of the included patients and omitted from the final model. All patients started in the state 'DLI1'. The number at the bottom left corner of the starting state shows the number of patients included in the model. The numbers at the bottom right corner of the boxes show the numbers of the patients who were in that state at the end of their follow-up. The numbers next to the arrows show the numbers of the patients who made that transition during their follow-up. The cGRFS is the sum of the probabilities of all green (thick border) states, the probability of being alive with GvHD the sum of all yellow (dashed border) states, the probability of death after start of tIS for GvHD the sum of all red (dotted border) states, the RFS is the sum of all green (thick border) and yellow (dashed border) states, and the OS the sum of all non-death states. For these summarizing measures, no distinction was made between states after the first DLI or after multiple DLIs.

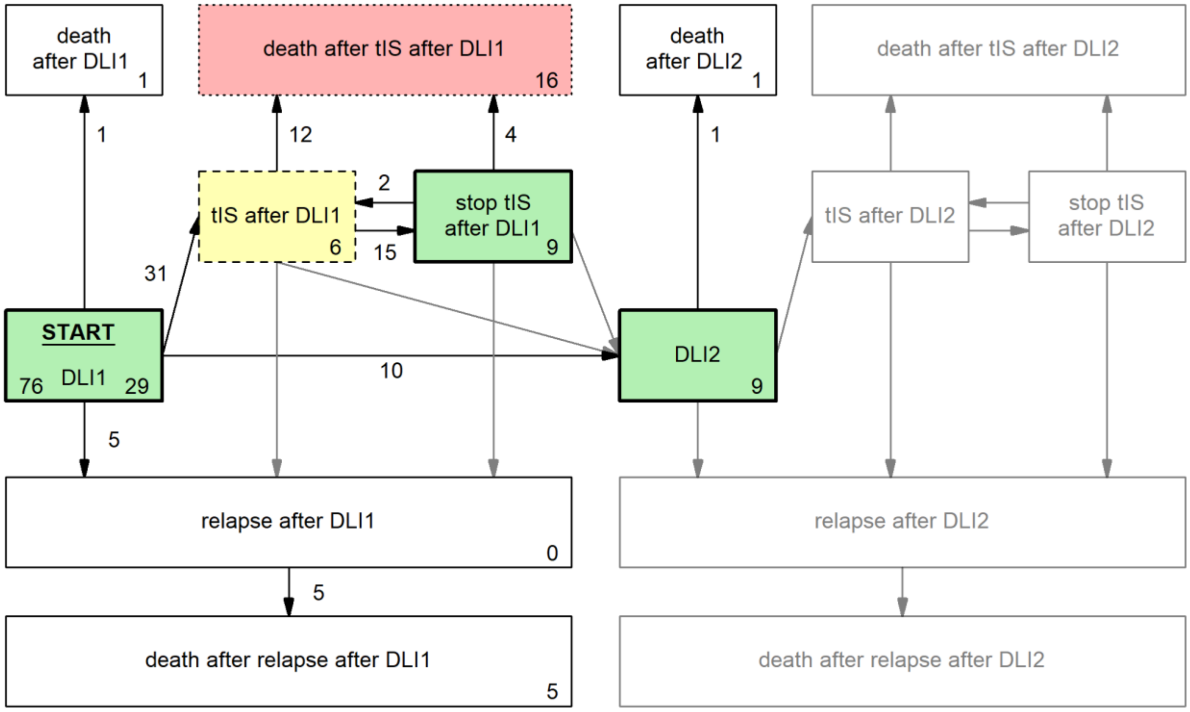

Supplementary Figure 2. Multi-state model for 6-month DLI. Boxes represent states and arrows represent the transitions between the states. Grey states and transitions were not used by any of the included patients and omitted from the final model. All patients started in the state 'DLI1'. The number at the bottom left corner of the starting state shows the number of patients included in the model. The numbers at the bottom right corner of the boxes show the numbers of the patients who were in that state at the end of their follow-up. The numbers next to the arrows show the numbers of the patients who made that transition during their follow-up. The cGRFS is the sum of the probabilities of all green (thick border) states, the probability of being alive with GvHD the sum of all yellow (dashed border) states, the probability of death after start of tIS for GvHD the sum of all red (dotted border) states, the RFS the sum of all green (thick border) and yellow (dashed border) states, and the OS the sum of all non-death states. For these summarizing measures, no distinction was made between states after the first DLI or after multiple DLIs.

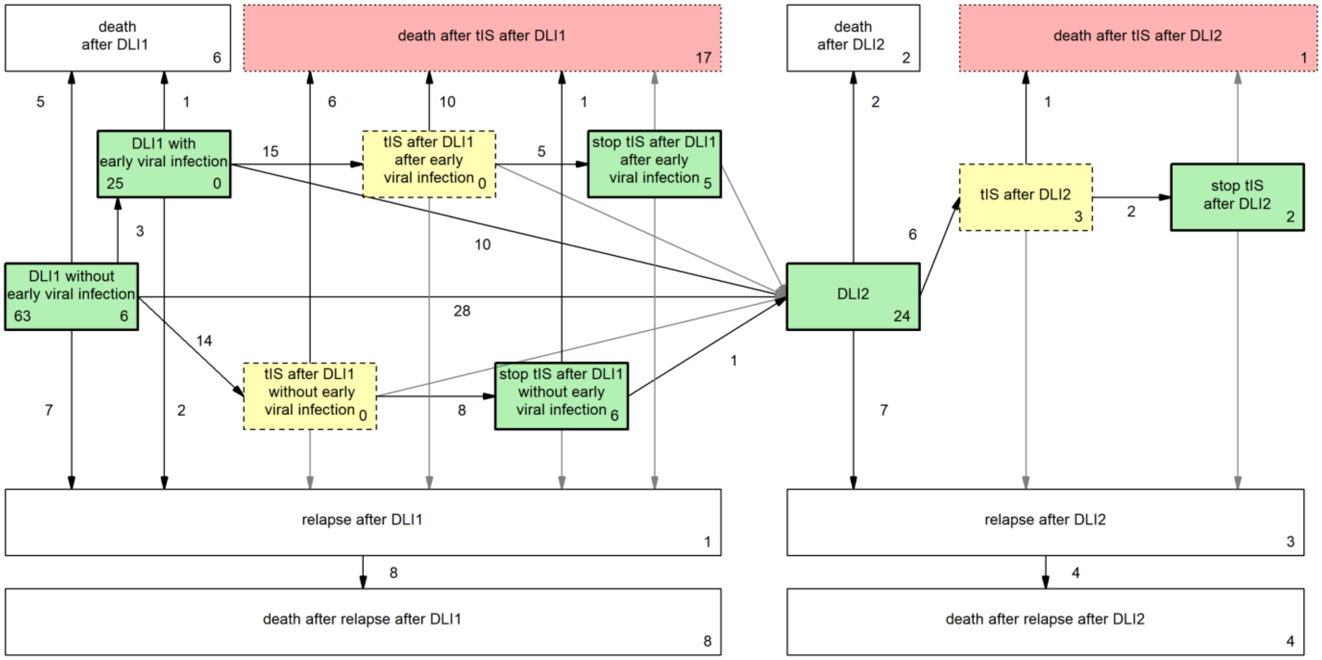

Supplementary Figure 3. Multi-state model for low-dose 3-month DLI considering early viral infections. Boxes represent states and arrows represent the transitions between the states. Grey transitions were not used by any of the included patients and omitted from the final model. 63 patients had no viral infection during the last week before DLI and started in the state ‘DLI1 without early viral infection’, while the 25 patients with a viral infection during the last week before DLI started in the state ‘DLI1 with early viral infection’ (see the numbers at the bottom left corner of the two starting states). Patients who had an early viral infection during the first 2 weeks after DLI without any prior event moved from ‘DLI1 without early viral infection’ to ‘DLI1 with early viral infection’ at time of the viral infection. The numbers at the bottom right corner of the boxes show the numbers of the patients who were in that state at the end of their follow-up. The numbers next to the arrows show the numbers of the patients who made that transition during their follow-up. The cGRFS is the sum of the probabilities of all green (thick border) states, the probability of being alive with GvHD the sum of all yellow (dashed border) states, the probability of death after start of tIS for GvHD the sum of all red (dotted border) states, the RFS the sum of all green (thick border) and yellow (dashed border) states, and the OS the sum of all non-death states. For these summarizing measures, no distinction was made between states after the first DLI or after multiple DLIs.

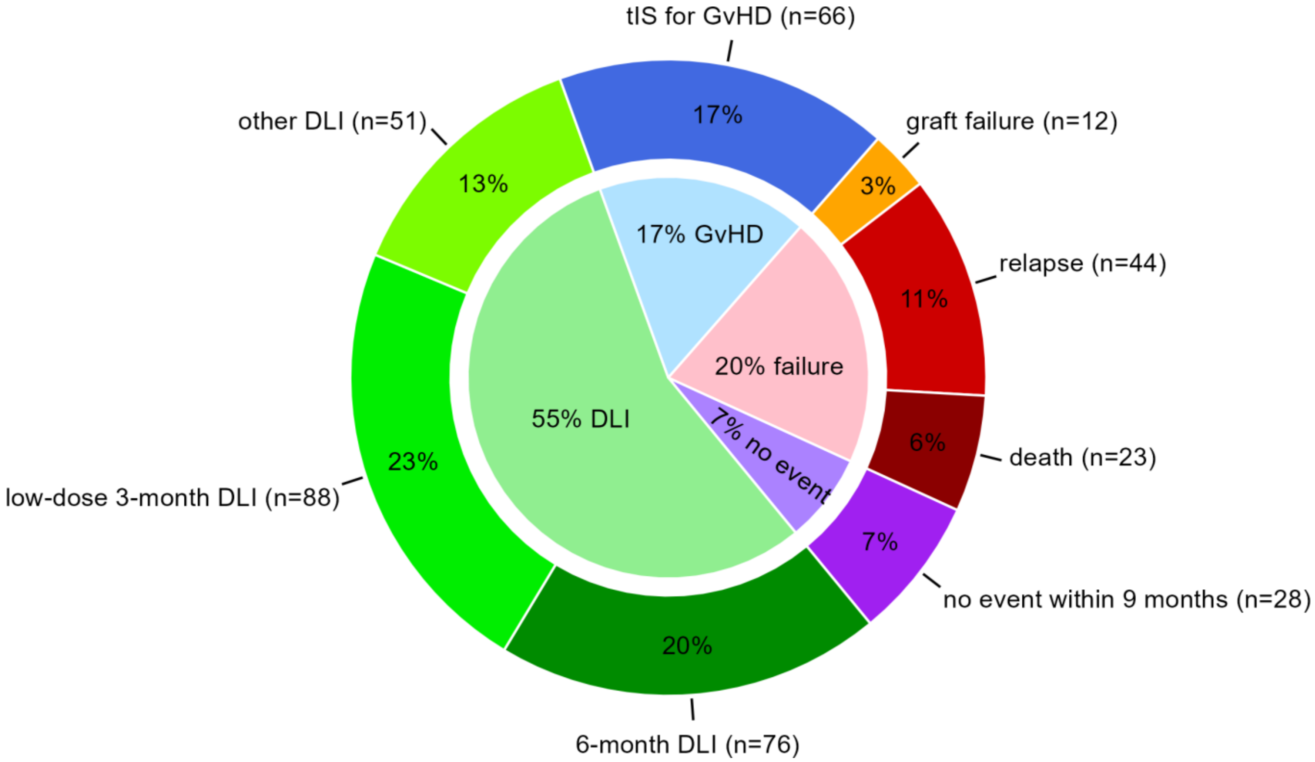

Supplementary Figure 4. Selection of the DLI cohorts. Events during the first 9 months after alloSCT for the total cohort. Per patient only the first occurring event was taken into account. The inner circle describes the main event categories (DLI, GvHD, treatment failure (i.e., death, relapse or graft failure), no event), while the outer circle further specifies the kind of DLI or treatment failure. The 88 patients who received the low-dose 3-month DLI and the 76 patients who received the 6-month DLI as first DLI were included in the DLI analyses.

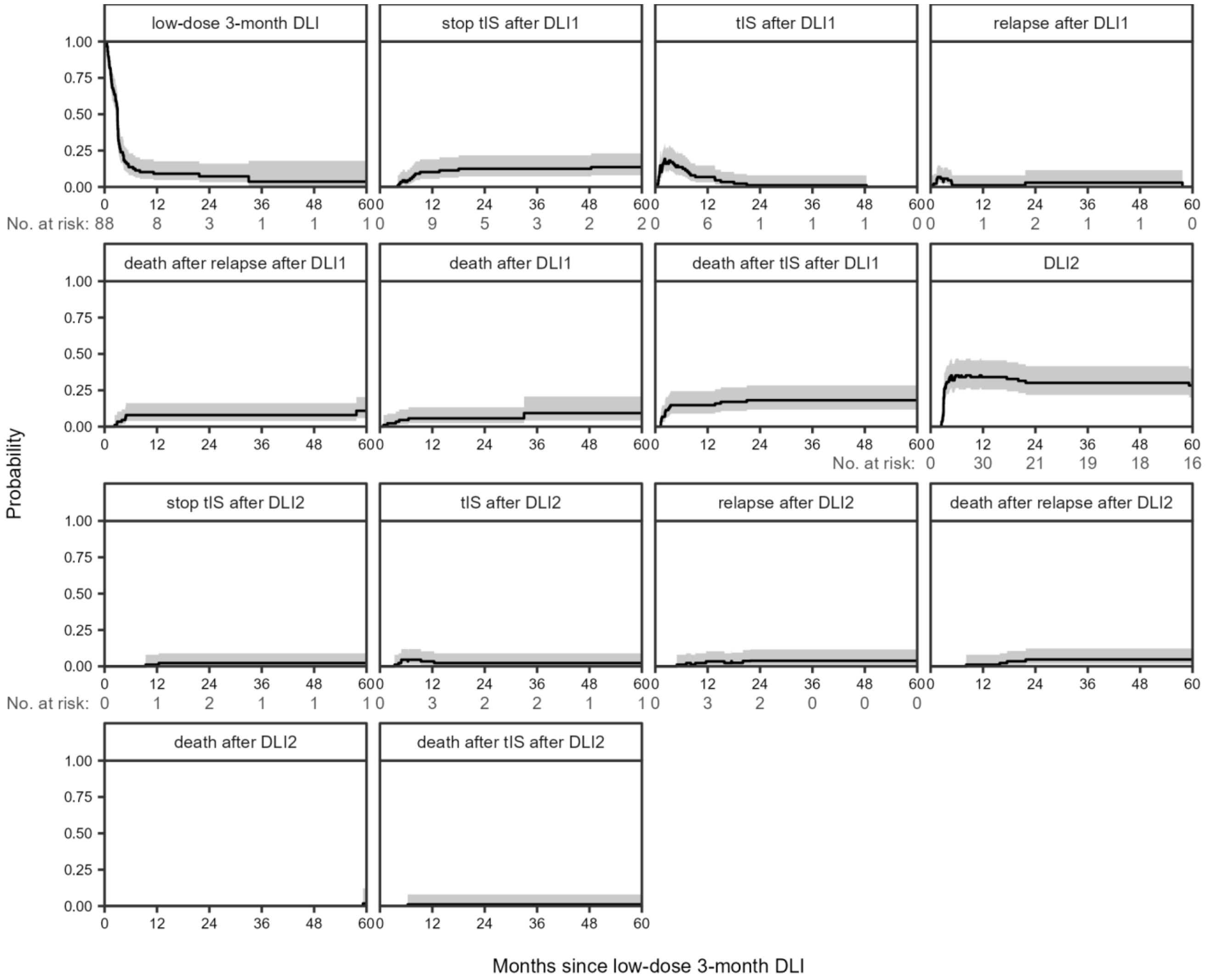

Supplementary Figure 5. Outcomes after low-dose 3-month DLI: probabilities with associated 95% confidence intervals per state. Probabilities with associated 95% confidence intervals for each state. The at risk numbers are shown for all non-death states and indicate the numbers of uncensored patients present in each state at different timepoints.

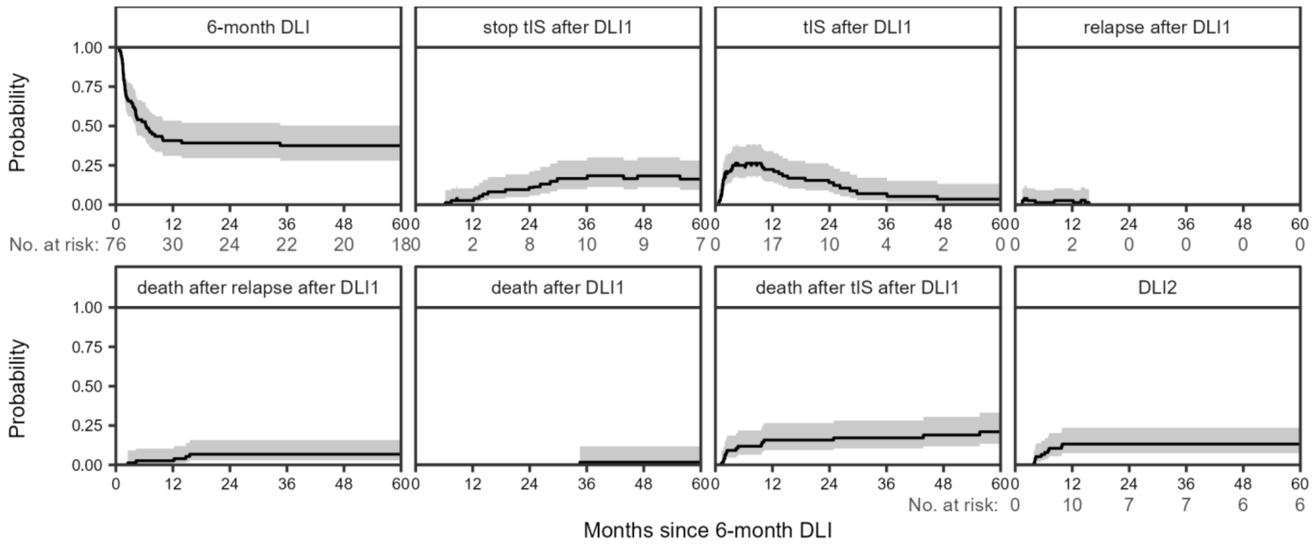

Supplementary Figure 6. Outcomes after 6-month DLI: probabilities with associated 95% confidence intervals per state. Probabilities with associated 95% confidence intervals for each state. The at risk numbers are shown for all non-death states and indicate the numbers of uncensored patients present in each state at different timepoints. Only states that were occupied within 5 years after 6-month DLI are shown.

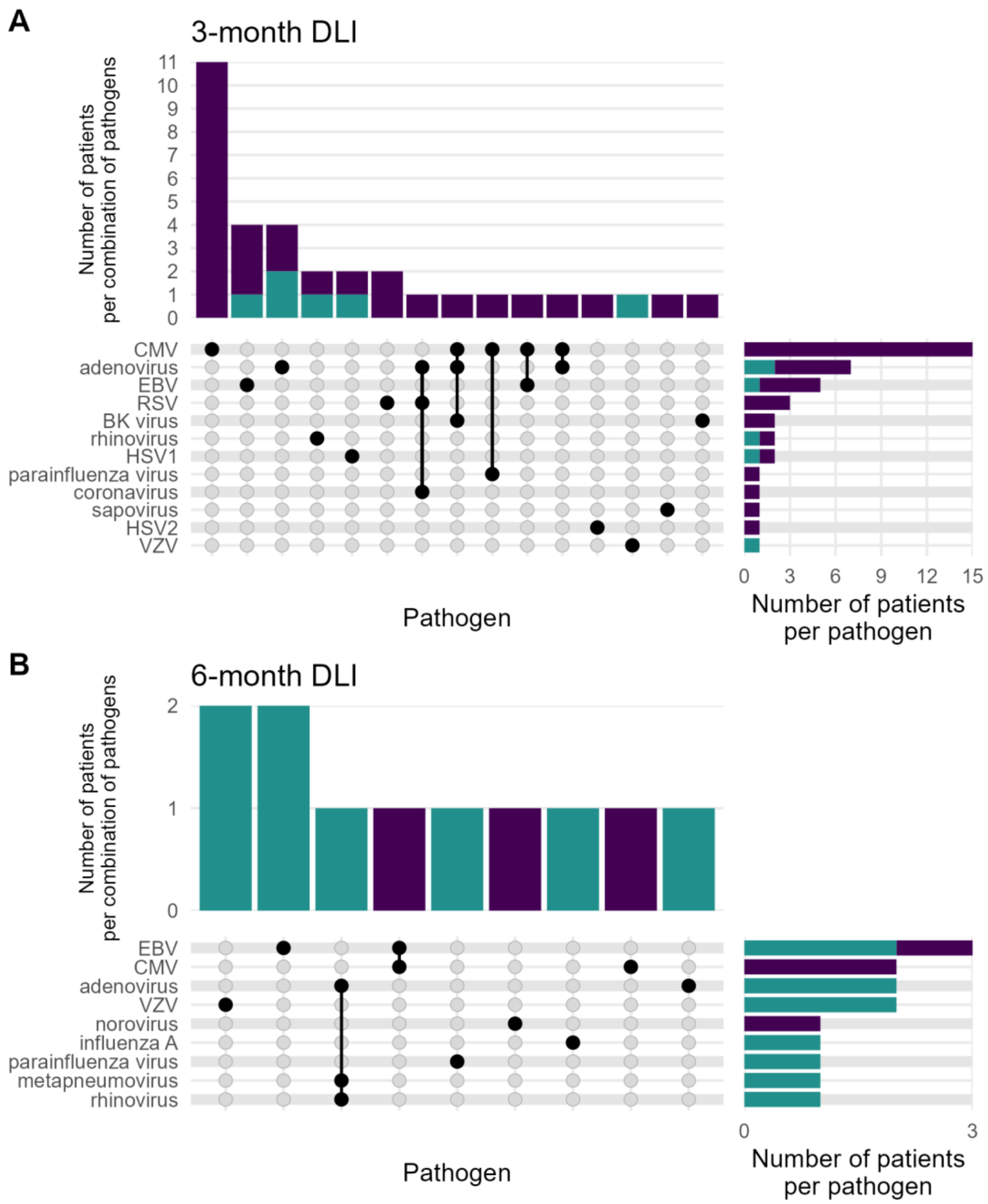

Supplementary Figure 7. Pathogens of viral infections close to the first DLI. UpSet plots of all viral pathogens present within 1 week before and 8 weeks after the low-dose 3-month DLI (panel A) or the 6-month DLI (panel B). The horizontal bar charts show for each of the pathogens the number of patients with this pathogen. As can be seen by the dot-connecting lines, some patients had multiple pathogens during this period. The vertical bar charts show the numbers of patients for each of the combinations. Purple indicates early onset (<2 weeks after DLI) viral infections, turquoise late onset (>2 weeks after DLI) infections. For instance, 3 patients had an EBV viremia close to the 6-month DLI, of whom two beyond 2 weeks after DLI without any other pathogen. The other patient had an early EBV viremia and a CMV viremia.

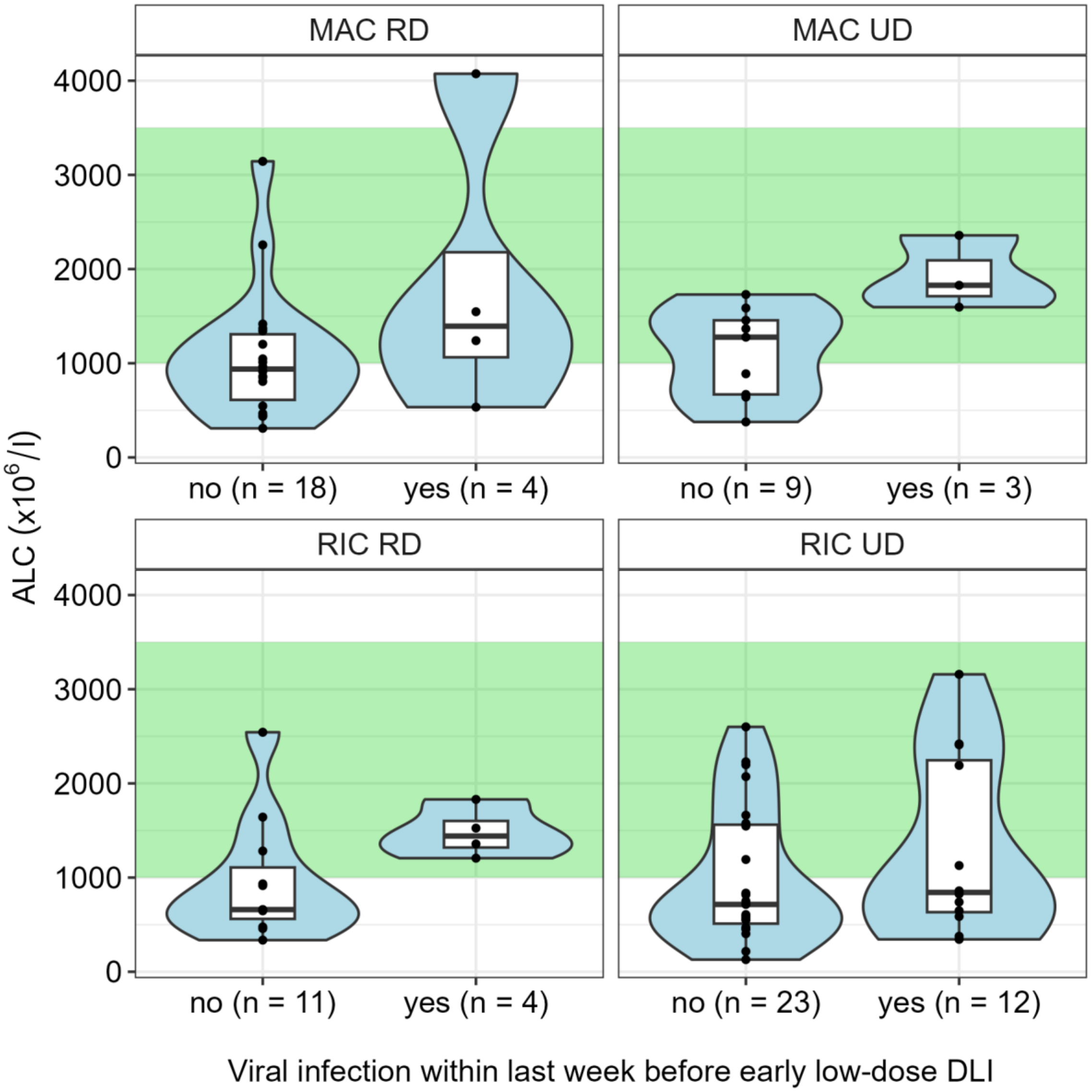

Supplementary Figure 8. ALC per conditioning/donor type and viral status at time of low-dose 3-month DLI. ALC at time of low-dose 3-month DLI per conditioning/donor type and the presence of a viral infection within the last week before this DLI. The boxplots are combined with violin plots showing the kernel probability density to visualize the distribution of the data. The lower and upper hinges of the boxplots correspond to the 25th and 75th percentiles. The green area shows the normal range used in our laboratory. Four patients for whom the exact ALC at time of DLI was unknown, were excluded (1 MAC RD without viral infection, 1 MAC RD with viral infection, 1 RIC UD without viral infection, 1 RIC UD with viral infection).

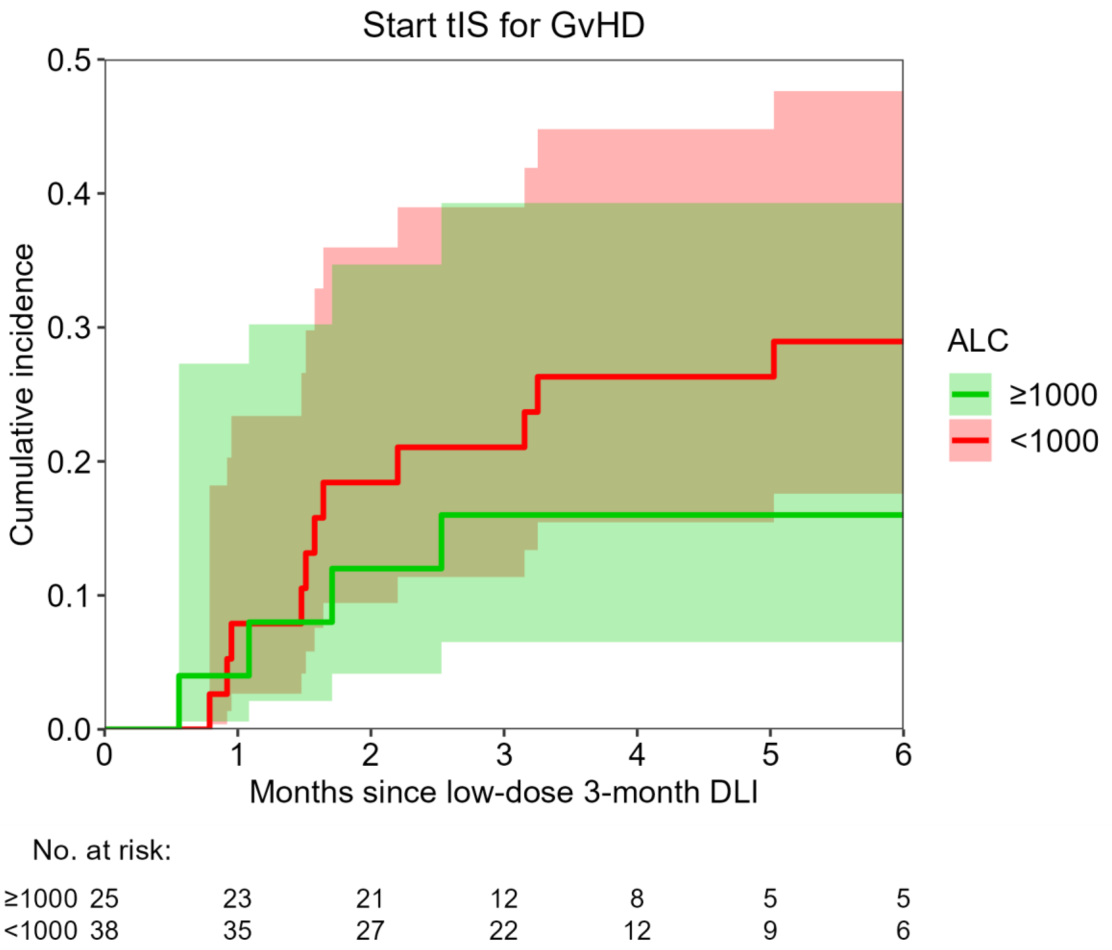

Supplementary Figure 9. Cumulative incidence of tIS for GvHD after low-dose 3-month DLI in the absence of viral infections within the last week before DLI. Cumulative incidences with associated 95% confidence intervals of tIS for GvHD after the low-dose 3-month DLI for patients with ALC ≥1000x106/l (n=25) or lower (n=38). This was calculated in a competing risks model starting at time of low-dose 3-month DLI with start tIS, relapse, death and DLI2 as competing events. The 25 patients with a viral infection during the last week before the low-dose 3-month DLI were excluded.

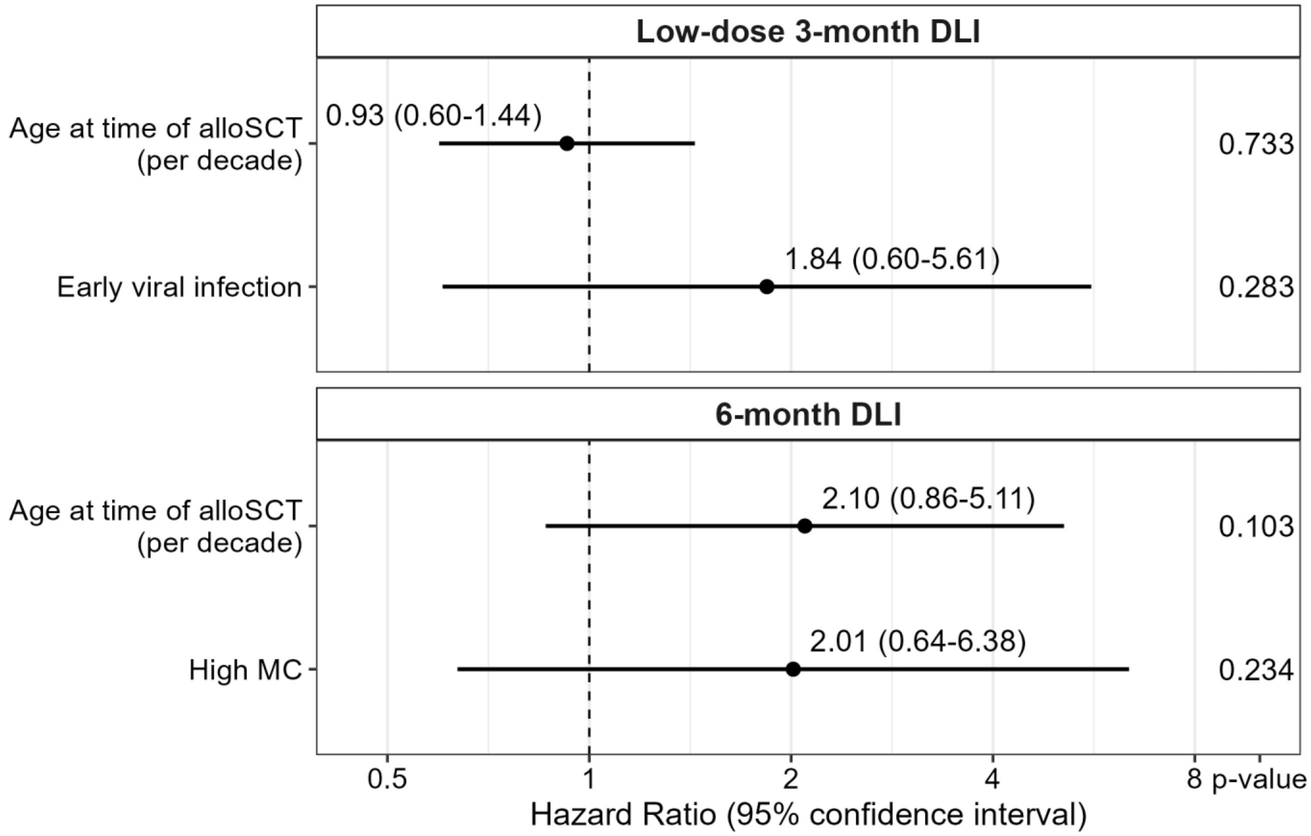

Supplementary Figure 10. Risk factors for death during tIS for GvHD. Cox proportional hazards models for the transition from tIS for GvHD after DLI1 to death (see Figure 1). Based on complete case analysis (n=29 for low-dose 3-month DLI and n=31 (age) or n=30 (chimerism) for 6-month DLI). DLI, donor lymphocyte infusion; alloSCT, allogeneic stem cell transplantation; high MC, ≥5% mixed chimerism in the bone marrow

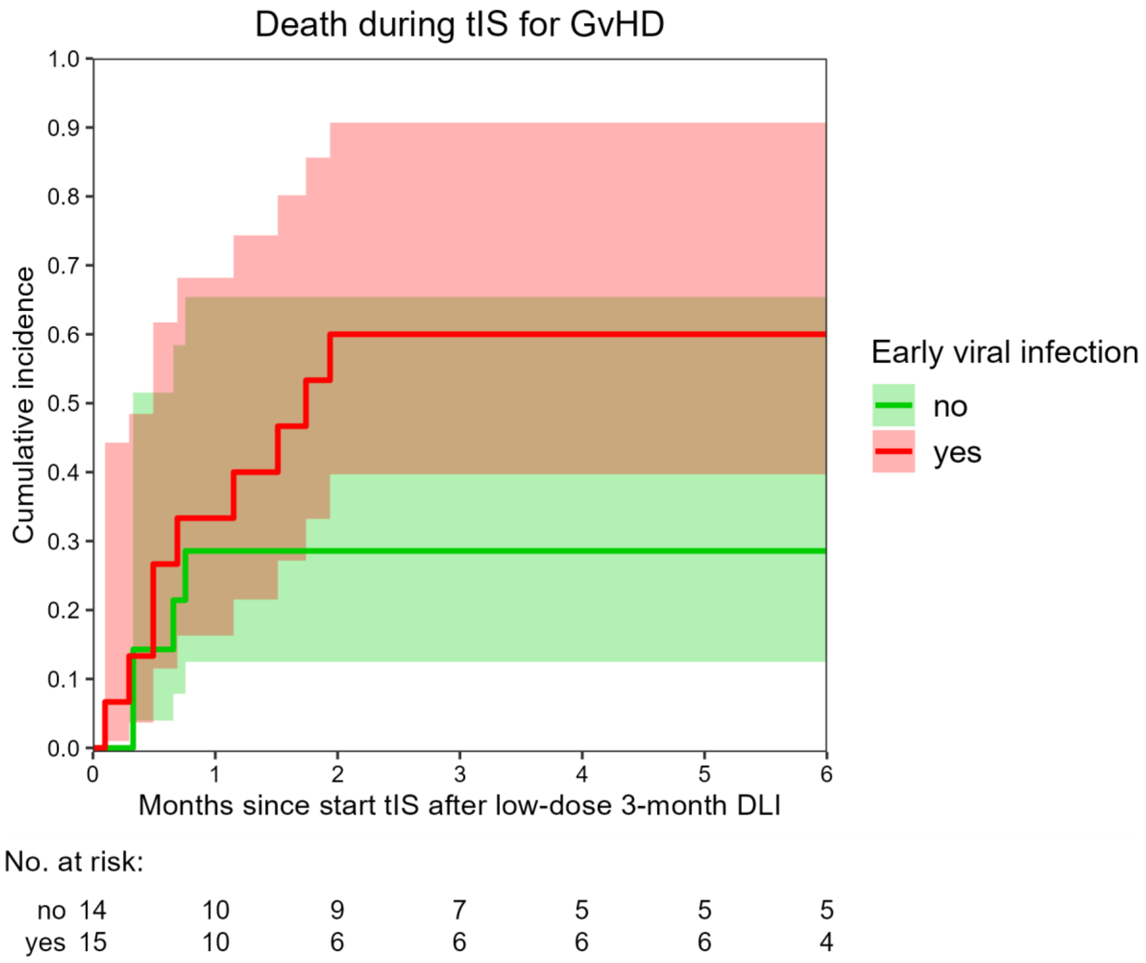

Supplementary Figure 11. Cumulative incidence of death during tIS for GvHD after low-dose 3-month DLI. Cumulative incidences with associated 95% confidence intervals of death during tIS for GvHD for patients who developed GvHD after an early viral infection and those without any early viral infection. This was calculated in a competing risks model starting at time of start tIS for GvHD after DLI with death, DLI2, relapse and stop tIS as competing events.

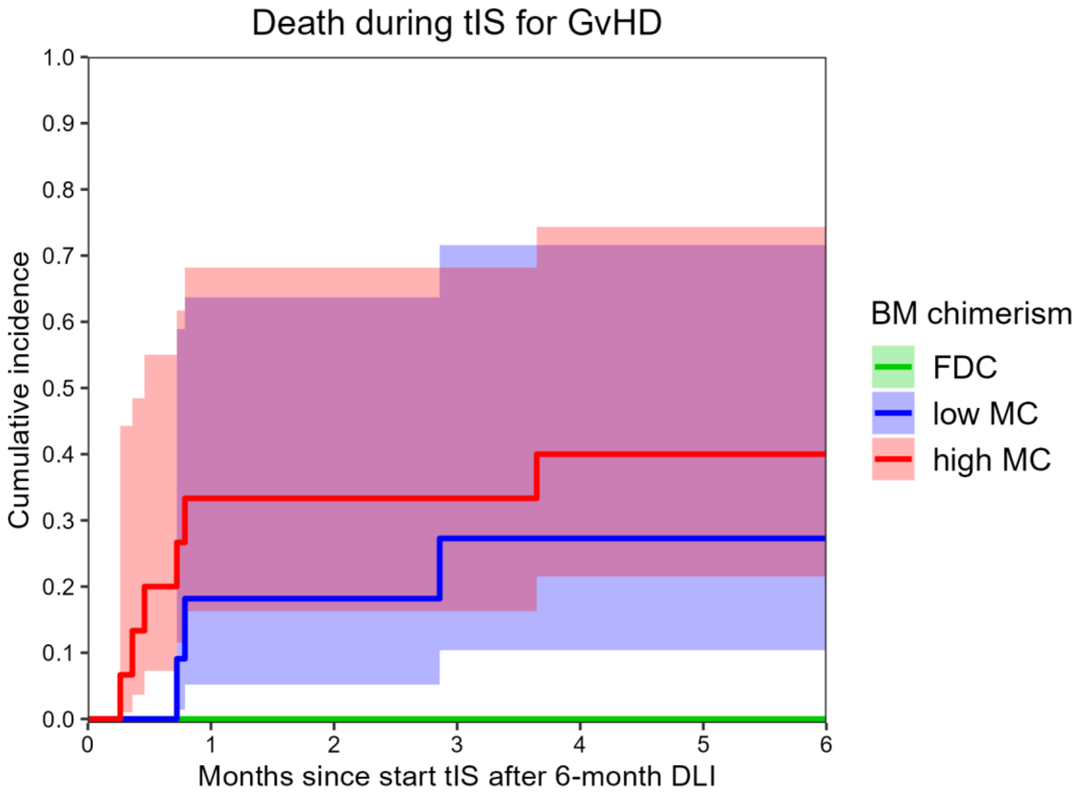

No. at risk:

FDC	6	6	6	6	5	5	4
low MC	11	9	9	8	8	8	7
high MC	15	10	10	10	9	9	9

Supplementary Figure 12. Cumulative incidence of death during tIS for GvHD after 6-month DLI. Cumulative incidences with associated 95% confidence intervals of death during tIS for GvHD per BM chimerism status at time of DLI for patients who developed GvHD after the 6-month DLI. This was calculated in a competing risks model starting at time of start tIS for GvHD after DLI with death, DLI2, relapse and stop tIS as competing events. One patient with FDC and one with high MC had two tIS episodes and entered the risk set twice.

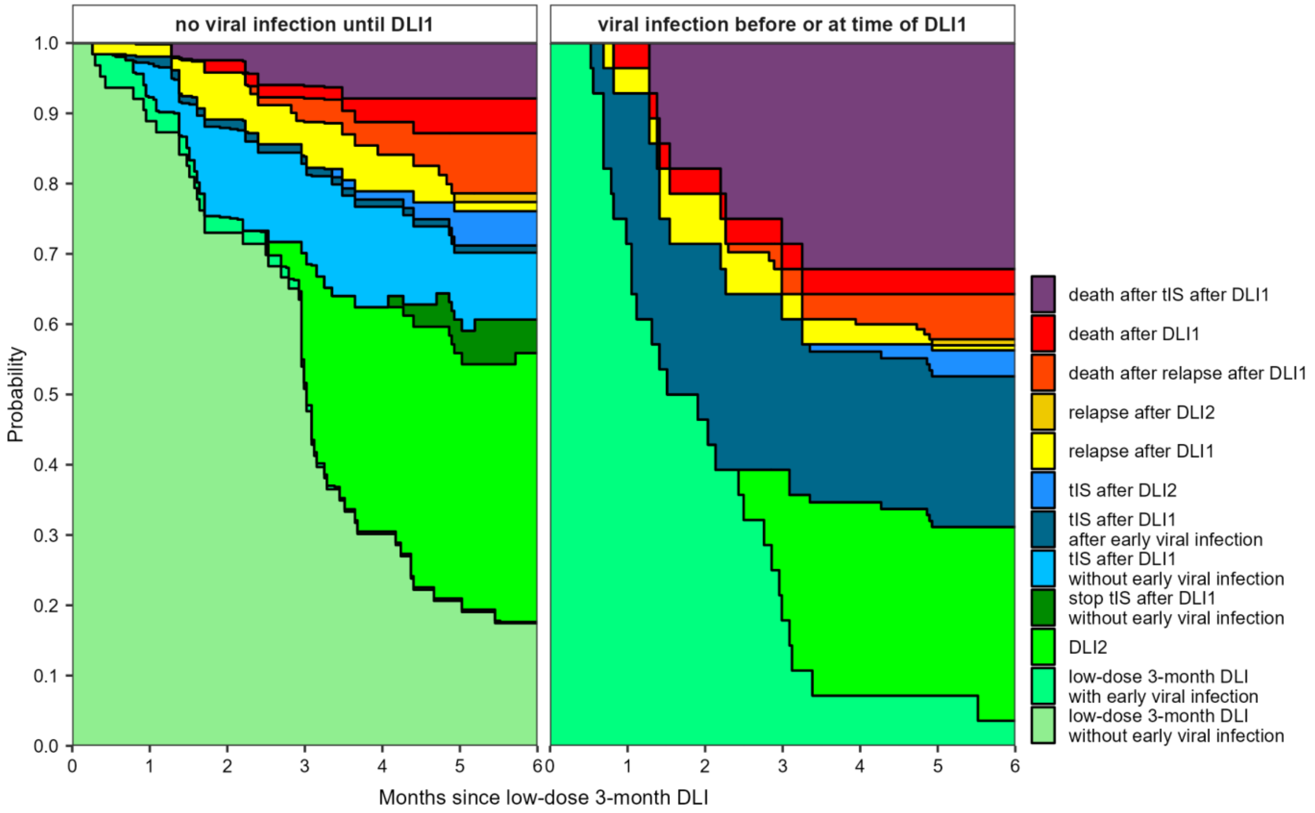

Supplementary Figure 13. Outcomes after low-dose 3-month DLI based on the viral status at time of DLI. Stacked state occupation probabilities after low-dose 3-month DLI based on the viral status at time of DLI (viral infection during the last week before DLI (n=25) or no viral infection until DLI (n=63). The estimates are based on the non-parametric multi-state model in **Supplemental Figure 3** which has two starting states ('DLI1 without early viral infection' and 'DLI1 with early viral infection'). The difference between two adjacent curves represents the probability of being in the corresponding state. States that were not used within 6 months after DLI were omitted from the legend.
